# Burden of falls among people aged 60 years and older in mainland China, 1990–2019: findings from the Global Burden of Disease Study 2019

**DOI:** 10.1016/S2468-2667(21)00231-0

**Published:** 2021-11-25

**Authors:** Pengpeng Ye, Yuliang Er, Haidong Wang, Lijie Fang, Bingqin Li, Rebecca Ivers, Lisa Keay, Leilei Duan, Maoyi Tian

**Affiliations:** a The George Institute for Global Health, UNSW Sydney, Sydney, NSW, Australia; b School of Population Health, UNSW Sydney, Sydney, NSW, Australia; c School of Optometry and Vision Science, UNSW Sydney, Sydney, NSW, Australia; d Faculty of Medicine and Health and Social Policy Research Centre, UNSW Sydney, Sydney, NSW, Australia; e National Centre for Non-Communicable Disease Control and Prevention, Chinese Centre for Disease Control and Prevention, Beijing, China; f Institute for Health Metrics and Evaluation, University of Washington, Seattle, WA, USA; g School of Sociology and Population Studies, Renmin University of China, Beijing, China; h The George Institute for Global Health at Peking University Health Science Centre, Beijing, China; i School of Public Health, Harbin Medical University, Harbin, China

## Abstract

**Background:**

Falls in older people have become a major public health concern worldwide, but a comprehensive assessment of the burden of falls for older people in mainland China has not been done. We aimed to investigate the burden of falls among older people at the national and subnational level in mainland China, and explore the trends from 1990 to 2019, using data from the Global Burden of Diseases, Injuries, and Risk Factors Study (GBD) 2019.

**Method:**

Using data from GBD 2019, we estimated the burden of falls among people aged 60 years and older by sex and age group in terms of incidence, mortality, and disability-adjusted life-year (DALY) rates and assessed these indicators at the subnational level in 31 geographical units (hereafter called provinces). We investigated the overall trend in the burden of falls across these 31 provinces from 1990 to 2019, and assessed the change in the burden of falls by sex, age group (60–64, 65–69, 70–74, 75–79, and ≥80 years), and province between 1990 and 2019.

**Findings:**

In 2019, in mainland China, the incidence rate of falls among people aged 60 years and older was 3799·4 (95% uncertainty interval [UI] 3062·4–4645·0) new falls per 100 000 population, and 39·2 deaths (21·8–48·8) per 100 000 population and 1238·9 DALYs (920·5–1553·2) per 100 000 population were due to falls. We found no significant difference in the burden of falls between males and females. The incidence, mortality, and DALY rates of falls for people aged 80 years and older were significantly higher than those in the other age groups, except for incidence rate, which was non-significantly different between the age 75–79 years group and the oldest age group. Large variations in the incidence and DALY rates of falls were observed across 31 provinces. Although between 1990 and 2019 we found no significant changes in overall mortality due to falls in all provinces and in DALY rates for 23 provinces (DALY rates significantly decreased in two provinces and increased in six provinces), we found large increases in the incidence rate of falls in both males (percentage change between 1990 and 2019: 82·9% [67·4–100]) and females (77·0% [63·3–91·8]). The percentage change in incidence rate of falls between 1990 and 2019 varied from 50·0% (42·2–59·5) for people aged 60–64 years to 123·8% (105·4–141·9) for people aged 80 years and older. All provinces had significant increases in the incidence rate of falls between 1990 and 2019, with Sichuan having the greatest increase (148·5% [125·5–171·4]) and Jilin the smallest increase (14·7% [3·6–26·1]).

**Interpretation:**

Between 1990 and 2019, the incidence rate of falls increased substantially in older adults across mainland China, whereas the rates of mortality and DALY of falls among older people remained relatively stable, suggesting improvements in outcomes of falls. Nevertheless, falls remain an ongoing health burden for older people in mainland China, and there is an urgent need to introduce system-wide, integrated, and cost-effective measures to protect and support older people to minimise their risks and combat an increasing absolute burden as the population continues ageing.

**Funding:**

Bill & Melinda Gates Foundation.

## Introduction

Falls are the second leading cause of unintentional injury deaths worldwide.^1^ Globally, approximately 684 000 people die from falls each year, of which over 80% occur in low-income and middle-income countries.^1^ Worldwide every year, 37·3 million severe falls occur that warrant medical attention, resulting in a substantial loss of more than 17 million disability-adjusted life-years (DALYs).^1^ People older than 65 years are more susceptible to fatal falls and other serious consequences, such as hip fractures.^2^

In mainland China, falls are the leading cause of injury-related mortality among people aged 65 years and older, and have been recognised as a complex but preventable health issue.^3^ Previous cross-sectional studies and systematic reviews have reported on the epidemiology of falls in older people in mainland China;^4^, ^5^, ^6^, ^7^, ^8^, ^9^ however, large variations in estimates of incidence, mortality, and DALYs due to falls in this population exist, mainly because of differences in the methods used to collect data, inconsistent definitions, variation in age groups and populations assessed, and heterogeneity in the study location (including community *vs* facility-based studies) and duration.^4^ No uniform national data are available on the burden of falls among people aged 60 years and older in mainland China over the past 30 years.^10^ Details of our literature search for publications on the epidemiology of falls among people aged 60 years and older in mainland China are in the appendix (p 1).

**Research in context**

**Evidence before this study**

Falls are an increasing health concern for older people in mainland China. We searched for publications in English and Chinese reporting the epidemiology of falls among older people in mainland China on Ovid MEDLINE and Embase from database inception until July 1, 2020, using the terms “fall”, “disease burden”, “mortality”, “incidence”, “prevalence”, “disability-adjusted life year”, epidemiology”,”epidemiological data”, “China”, “Chinese”, “aged”, “old”, AND “elderly”. Most previous studies described the incidence or prevalence rates of falls for older people from specific provinces of China on the basis of cross-sectional surveys or meta-analyses. One study reported the DALYs of falls among people aged 70 years and older at the national level using Global Burden of Disease Study 2013 data, while one study reported the trend of the mortality rate of falls among people aged 65 years and older from 2006 to 2016 at the national level. However, to date, no study has systematically measured the burden of falls in terms of incidence, mortality, and DALY rates in people aged 60 years and older both at the national and subnational level in mainland China from 1990 to 2019.

**Added value of this study**

We systematically and comprehensively measured the burden of falls in terms of incidence, mortality, and DALY rates among people aged 60 years and older, by sex, across 31 subnational units (ie, provinces) of mainland China in 2019 and examined the trends of these outcome indicators over the past three decades. We found a substantial increase in the incidence rate of falls regardless of sex, age group, or geographical location. We found minor sex disparities in incidence and mortality rates. We found that the highest risk group was males aged 80 years and older. We also found a large variation in incidence and DALY rates of falls across provinces.

**Implications of all the available evidence**

If unmitigated, the burden of falls has potential to grow further in mainland China as the population ages. Based on our findings, we make three recommendations. First, programmes for the prevention of falls need to be implemented, and are particularly crucial for the oldest-old age group. Second, programmes on prevention of falls need to be equitably administered, but are particularly important for the oldest-old people. Finally, more efforts and resources need to be allocated to aid prevention of falls among older people at the national and provincial level, particularly for five provinces in the southwest and southeast coastal areas of mainland China where incidence and overall burden are high and increasing.

A comprehensive and coherent measurement framework incorporating transparent data sources, standardised data processing, and up-to-date statistical synthesis approaches were provided by the Global Burden of Diseases, Injuries, and Risk Factors Study (GBD) 2019 to quantify health loss due to 369 diseases and injuries and 87 risk factors across 204 countries and territories from 1990 to 2019.^11^, ^12^, ^13^ Here, we aimed to comprehensively measure the spatiotemporal variation of the burden of falls among people aged 60 years and older in mainland China, at both national and subnational levels, over the past three decades, based on GBD 2019. This manuscript was produced as part of the GBD Collaborator Network and in accordance with the GBD Protocol.

## Methods

### Overview

In this analysis, we investigated the trend in incidence, mortality, and DALY rates of falls among people aged 60 years and older in 31 geographical units in mainland China and the change in these three indicators in the same population by sex, age group, and province between 1990 and 2019. The geographical units of analysis are provincial-level jurisdictions including 22 provinces, five autonomous regions, and four municipalities directly under the Chinese Government. Hong Kong, and Macau, and Taiwan (province of China) were not included in the analysis. All 31 geographical units are referred to as provinces hereafter. We also designated the different provinces as being in the east, middle, west, and northeast of mainland China using the definitions of the Chinese National Bureau of Statistics of China.^14^

The measurement framework and estimates from GBD 2019 covering 204 countries and territories from 1990 to 2019 are described in the GBD 2019 capstone publications.^11^, ^12^, ^13^ The methods and data used for estimation of the burden of injury morbidity and mortality are provided in detail in the GBD 2017 literature.^3^, ^15^, ^16^ The key features and new updates for injury-specific data processing in GBD 2019 are summarised here.

This study has been registered at the Scientific Publications Team of the Institute for Health Metrics and Evaluation (IHME), University of Washington (ID: 1180-GBD2019-032020). This study complies with the Guidelines for Accurate and Transparent Health Estimates Reporting (known as GATHER) statement,^17^ with further information provided in the appendix (p 2). All data used in this study were aggregated data and did not contain any individually identifiable information; therefore, no ethics approval or consent to participate are needed.

### Key definitions

In this analysis, we defined older people as people aged 60 years and older on the basis of the legal definition in China.^18^ In GBD 2019, falls were one of 30 mutually exclusive and collectively exhaustive external causes of injury categories.^11^ Falls were defined as a sudden movement downward due to slipping, tripping, or other unintentional movement that results in a person coming to rest inadvertently on the ground, floor, other lower level, or against an object resulting in death, disability, or tissue damage.^11^, ^16^, ^19^ The corresponding codes of the International Classification of Disease (ICD) are E880 to E886.99, E888 to E888.9, and E929.3 in the ninth edition (ICD-9) and W00 to W19.9 in the tenth edition (ICD-10).^11^, ^16^, ^19^ The potential disabling outcomes that can occur after a fall include 47 mutually exclusive and collectively exhaustive nature-of-injury categories (eg, fractures, head injuries, and dislocations), which are specified within chapters S and T in ICD-10 with codes 800 to 999 in ICD-9 (for more details on how these categories are used in GBD see the GBD compare tool).^11^ In GBD 2019, inpatient injuries were defined as injuries that led to overnight stay in hospital and outpatient injuries were defined as treatments that occurred in the outpatient settings or emergency care. Short-term injuries were defined as injuries lasting less than 1 year, and long-term injuries were defined as those lasting 1 year or longer, at which point we assume lifelong disability.^11^, ^12^, ^13^, ^15^

### Injury mortality and years of life lost

The estimation of injury mortality and years of life lost (YLLs) involved several steps. First, we mapped all usable mortality data with different versions of ICD codes to the GBD causes of injuries list. We also redistributed ill-defined causes of deaths to the injury cause list. Second, we used the Cause of Death Ensemble model (CODEm) framework to generate different submodels on the basis of the recommended covariates. Third, we portioned the datasets into two sections to train and test the submodels, using different proportions of each dataset to test and train each specific model, and we developed an ensemble model out of the submodels. Fourth, we determined the best-performing model on the basis of the out-of-sample predictive validity among the ensemble model and submodels. Fifth, we corrected the injury-specific deaths to ensure internal consistency. Sixth, we calculated the rate of injury-specific deaths on the basis of the GBD populations. Finally, we calculated YLLs as the product of injury-specific death rates and residual life expectancy at the age of death.^11^, ^12^, ^13^, ^15^

### Incidence and years lived with disability

To estimate the incidence of non-fatal outcomes, including years lived with disability, first we used a Disease Model-Bayesian Meta-Regression (DisMod-MR; version 2.1) to model and infer the incidence of each cause of injury warranting medical care by sex, age group (age 60–64, 65–69, 70–74, 75–79, and ≥80 years), year, and province. Second, we derived the outpatient incidence from the estimated incidence using the outpatient coefficient. We then adjusted the incidence by removing injury deaths from the incidence pool. Third, we generated cause–nature matrices on the basis of dual-coded clinical data and separately calculated the age-specific, sex-specific, year-specific, and province-specific incidence of each cause–nature combination. Fourth, we converted these incidence estimates into short-term and long-term injury incidence estimates. We used DisMod-MR to convert the long-term injury incidence into long-term prevalence for each cause–nature combination. The short-term incidence was multiplied by the duration of injury to generate short-term prevalence for each cause–nature combination. Fifth, we calculated short-term disability weights and multiplied them by the short-term prevalence to generate short-term years lived with disability (YLDs). Sixth, we calculated the long-term prevalence on the basis of a similar process and adjusted using comorbidity correction. Finally, we aggregated the short-term and long-term YLDs across natures of injury for each cause by sex, age group, year, and province.^11^, ^12^, ^13^, ^15^

### Disability-adjusted life-years

In GBD 2019, DALYs were calculated as the sum of YLDs and YLLs for each cause by sex, age group, year, and province. One lost year of health was equivalent to one DALY. The gap between the current health status and full health situation (ie, the entire population living to an advanced age without disease and disability) was reflected by the sum of all DALYs across the population.^11^, ^12^, ^13^, ^15^

### Uncertainty intervals

We calculated uncertainty using the same methods described previously in GBD 2019.^11^, ^12^, ^13^, ^15^ Generally, in every step of the modelling process, we pulled 1000 draws from the distribution of each model component (eg, incidence, prevalence, proportion, case fatality, and disability weight) and used them to estimate the uncertainty.^11^, ^12^, ^13^, ^15^ We determined the distributions from the sampling error from data inputs, the uncertainty of the coefficients from DisMod-MR, and the uncertainty of severity distributions and disability weights.^3^, ^11^, ^12^, ^13^, ^15^ We summarised the final results as the mean of all draws.^11^, ^12^, ^13^, ^15^ We report the 95% uncertainty interval (UI) as the 25th and 975th ordered draw of the uncertainty distribution.^11^, ^12^, ^13^, ^15^ If the uncertainty intervals of two point estimates did not overlap then they were determined to be significantly different.^11^, ^12^, ^13^, ^15^ And if the 95% UI of a percentage change did not cross zero, it was determined to be a significant change.

### Updates to the estimation of the burden of falls in GBD 2019

A comparison of the updated methods used in GBD 2019 with those used in previous rounds of GBD has been described previously.^11^, ^12^, ^13^ There were four methodological changes specific to the estimation of the burden of falls. First, we used age-specific ratios between outpatients and inpatients, replacing a single outpatient coefficient in meta-regression Bayesian, regularised, trimmed (MR-BRT) analyses.^11^, ^19^ Second, we incorporated location-specific adjustments for access to health care.^11^, ^19^ Third, we used excess mortality rates of falls to stabilise geographical variation in spatiotemporal Gaussian process regression (ST-GPR) analyses.^11^, ^19^ Finally, we included new national survey data from the WHO study on Global Ageing and Adult Health pertaining to the Chinese population in the estimation of injuries due to falls.^11^, ^19^ The data sources we used to estimate the disease burden in China are listed on the Global Health Data Exchange, and the data sources closely related to the estimation of the burden of falls are provided in the appendix (p 3).^20^

### Role of the funding source

The funder of the study had no role in the study design, data collection, data analysis, data interpretation, or writing of the report.

## Results

In 2019, among people aged 60 years and older in mainland China, 3799·4 new falls (95% UI 3062·4–4645·0) per 100 000 population occurred, the mortality rate due to falls was 39·2 deaths (21·8–48·8) per 100 000 population, and the DALY rate for falls was 1238·9 DALYs (920·5–1553·2) per 100 000 population (table 1). Overall, we found no significant differences between older males and females for each of these three indicators (table 1). Among different age groups, for both males and females, the rates of incidence, mortality, and DALYs for people aged 80 years and older were significantly higher than those in the age 60–64, 65–69, 70–74, and 75–79 years groups; however, the difference was not significant for the DALY rate for males aged 75–79 years and aged 80 years and older (table 1). We found no significant differences between males and females for these outcomes by age group, except for rates of mortality due to falls in the age 60–64 years group, for which males had a significantly higher mortality rate than did females (table 1).

**Table 1:** Incidence, mortality, and DALY rates of falls among older people aged 60 years and older in mainland China in 2019, by sex and age groups

		**Females**			**Males**			**Total**		
		Incidence rate, per 100 000 population	Mortality rate, per 100 000 population	DALY rate, per 100 000 population	Incidence rate, per 100 000 population	Mortality rate, per 100 000 population	DALY rate, per 100 000 population	Incidence rate, per 100 000 population	Mortality rate, per 100 000 population	DALY rate, per 100 000 population
Age group, years										
	60–64	2401·5 (1535·8 to 3397·4)	4·7 (2·6 to 6·2)	524·0 (400·3 to 675·8)	2553·3 (1628·7 to 3620·5)	13·9 (7·6 to 19·2)	901·8 (647·4 to 1146·1)	2477·8 (1580·0 to 3514·7)	9·3 (5·5 to 12·2)	713·8 (539·0 to 900·2)
	65–69	2833·4 (1898·2 to 4051·5)	8·0 (4·2 to 10·4)	692·1 (519·9 to 884·3)	2646·8 (1744·3 to 3821·3)	17·2 (9·1 to 23·8)	1010·1 (732·5 to 1290·3)	2741·8 (1822·2 to 3945·5)	12·5 (7·4 to 16·2)	848·2 (642·9 to 1077·2)
	70–74	3494·5 (2273·9 to 4901·3)	16·5 (8·4 to 21·1)	976·2 (722·8 to 1263·3)	3092·7 (2026·8 to 4317·6)	28·7 (14·6 to 39·7)	1275·3 (918·5 to 1632·6)	3298·6 (2151·9 to 4597·8)	22·5 (12·6 to 28·6)	1122·0 (827·9 to 1424·0)
	75–79	5470·0 (3528·5 to 8076·5)	39·1 (18·8 to 50·1)	1489·9 (1059·6 to 1908·4)	4217·4 (2775·1 to 6219·8)	54·0 (28·5 to 74·0)	1701·7 (1180·9 to 2176·8)	4876·7 (3202·0 to 7195·2)	46·1 (25·6 to 57·8)	1590·2 (1172·3 to 2001·2)
	≥80	10 978·3 (8209·7 to 14 389·5)	201·6 (93·9 to 262·3)	3468·6 (2397·9 to 4294·2)	6971·3 (5064·5 to 9349·1)	193·0 (104·9 to 254·3)	3162·4 (2139·6 to 3992·2)	9419·2 (7007·4 to 12418·4)	198·3 (104·2 to 249·5)	3349·5 (2356·8 to 4128·9)
Total		4264·9 (3474·5 to 5166·7)	39·1 (18·9 to 50·5)	1172·9 (854·5 to 1481)	3294·6 (2618·0 to 4064·9)	39·3 (21·9 to 52·6)	1310·6 (937·6 to 1649·3)	3799·4 (3062·4 to 4645·0)	39·2 (21·8 to 48·8)	1238·9 (920·5 to 1553·2)

Data in parentheses are 95% uncertainty intervals. DALY=disability-adjusted life-year.

Across all provinces, we found large variations in incidence rates for older people, with the highest incidence rate being in Zhejiang (7170·9 new falls [95% UI 5835·3–8649·8] per 100 000 population), followed by Shanghai (6062·5 [4981·0–7266·8] per 100 000 population) and Fujian (5990·7 [4857·6–7249·2] per 100 000 population), while the lowest incidence rate was in Jilin (1892·0 [1449·5–2402·4] per 100 000 population), followed by Gansu (1976·3 [1564·7–2451·5] per 100 000 population) and Heilongjiang (1985·6 [1553·8–2482·4] per 100 000 population; appendix pp 4–9, 15). The mortality rates varied from a low of 11·14 deaths (95% UI 7·3–22·1) per 100 000 population in Jilin to a high of 101·6 deaths (20·9–145·3) per 100 000 population in Fujian (appendix pp 4–9, 16). However, we mostly found no significant difference in mortality rates across the 31 provinces. We found significant differences in DALY rates across about half of the 31 provinces. The three provinces with the highest DALY rates were Zhejiang (2258·7 [1278·7–2932·1] per 100 000 population), Fujian (2245·7 [1158·6–2918·0] per 100 000 population), and Yunnan (2113·1 [1221·5–2718·0] per 100 000 population); while the three provinces with the lowest DALY rates were Jilin (547·0 [402·1–738·5] per 100 000 population), Heilongjiang (596·4 [440·4–802·3] per 100 000 population), and Inner Mongolia (616·1 [458·6–820·5] per 100 000 population; appendix pp 4–9, 17). We also found no significant differences in incidence rates, mortality rates, and DALY rates between older males and females in each province, except for significantly different incidence rates between older males and females in Fujian, Guangdong, and Shanghai (appendix pp 4–6). In all provinces, the three indicators for burden of falls for people aged 80 years and older were significantly higher than for the age 60–64, 65–69, and 70–74 years groups, but not for all provinces for the age 75–79 years group (appendix pp 7–9).

**Figure 1:**
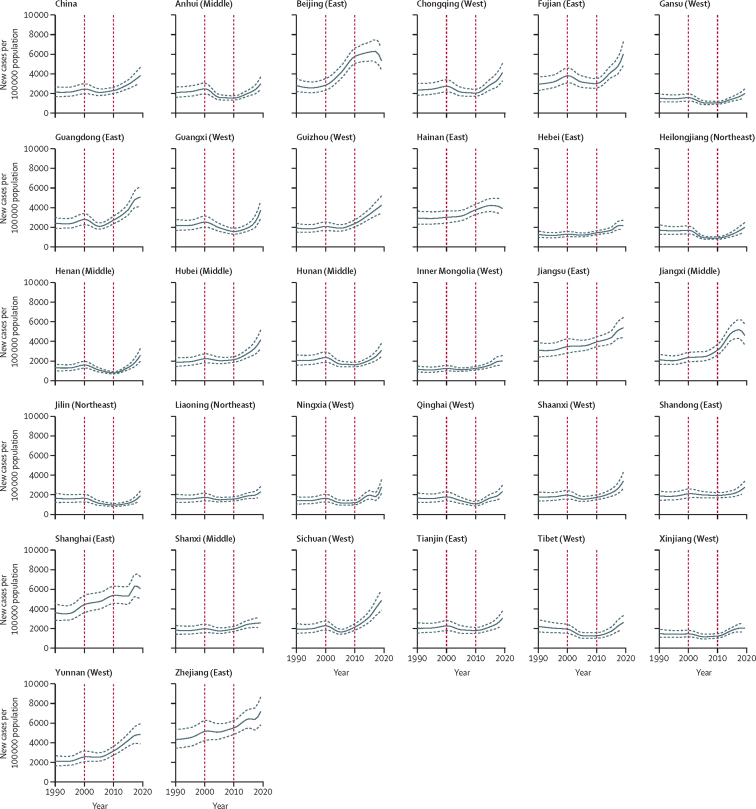
Change in incidence rate of falls among people aged 60 years and older for mainland China overall and in 31 provinces, 1990 to 2019 The red vertical lines indicate the years 2000 and 2010. The solid line shows the point estimate of number of new cases, with dashed lines showing the 95% uncertainty intervals.

**Figure 2:**
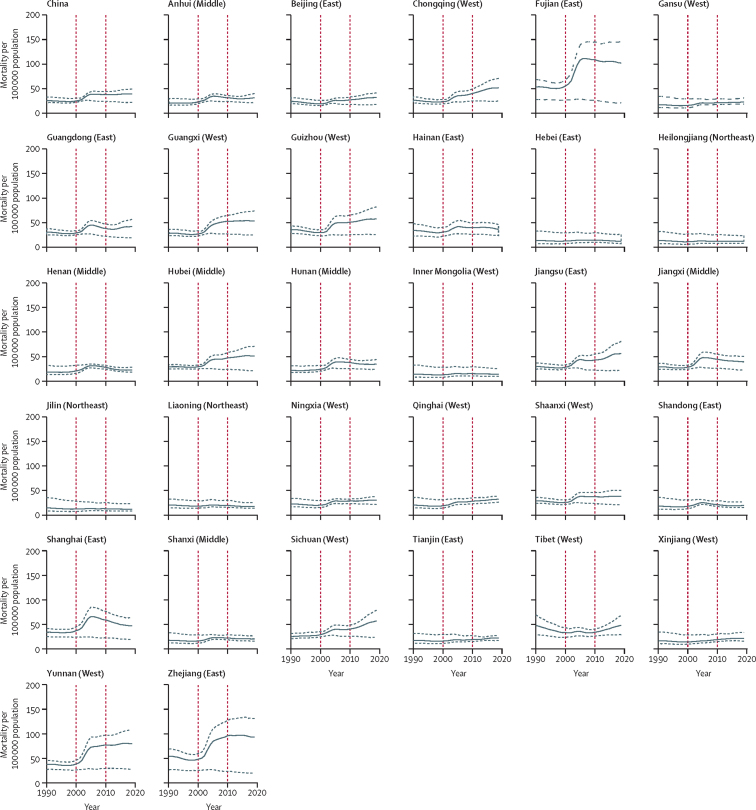
Change in mortality rate of falls among people aged 60 years and older for mainland China overall and in 31 provinces, 1990 to 2019 The red vertical lines indicate the years 2000 and 2010. The solid line shows the point estimate of mortality rate, with dashed lines showing the 95% uncertainty intervals.

**Figure 3:**
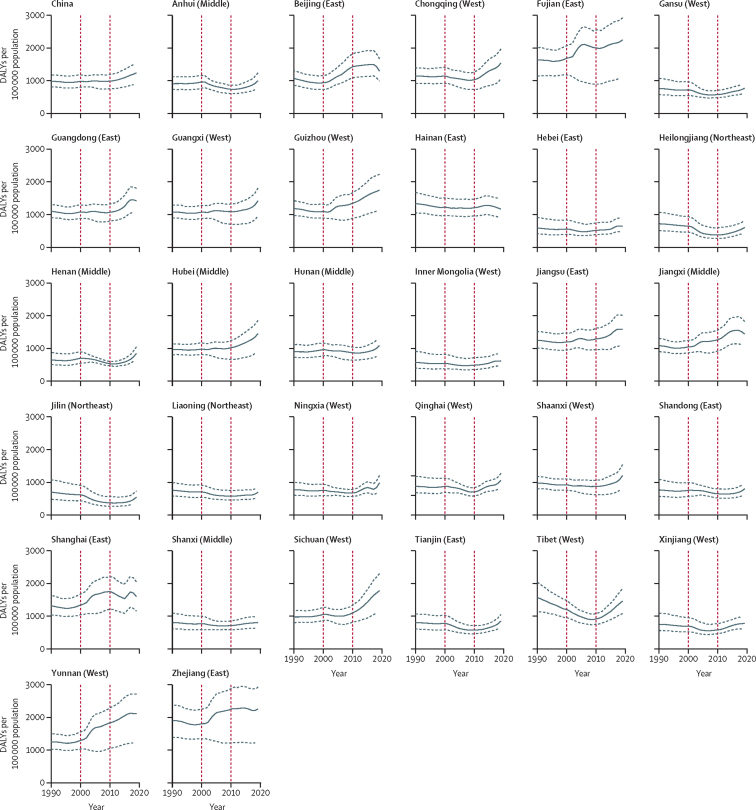
Change in DALY rate for falls among people aged 60 years and older for mainland China overall and in 31 provinces, 1990 to 2019 The red vertical lines indicate the years 2000 and 2010. The solid line shows the point estimate of DALY rate, with dashed lines showing the 95% uncertainty intervals. DALY=disability-adjusted life-year.

The incidence rate of falls for people aged 60 years and older in mainland China was relatively stable from 1990 to 2010, with a gradual increase from 2010 to 2019 (figure 1). Over the past three decades, the incidence rate of falls has increased by 79·2% (95% UI 65·92 to 93·7; table 2). There was a stable trend for the mortality rate from 1990 to 2000, followed by an increase in 2003, reaching a plateau between 2003 and 2019 (figure 2). Between 1990 and 2019, the percentage change in mortality rate was not significant (59·2% [–24·7 to 116·4]; table 2). The trend in DALY rates for falls was similar to that of incidence rate, but with no significant change between 1990 and 2019 (26·6% [–5·3 to 46·8]; figure 3, table 2).

**Table 2:** Percentage change in incidence, mortality, and DALY rates of falls among people aged 60 years and older in mainland China between 1990 and 2019, by sex and age group

		**Females**			**Males**			**Total**		
		Incidence rate	Mortality rate	DALY rate	Incidence rate	Mortality rate	DALY rate	Incidence rate	Mortality rate	DALY rate
Age group, years										
	60–64	47·0% (38·1 to 58·1)	−20·8% (−59·0 to 15·5)	−5·6% (−21·2 to 5·7)	52·9% (44·7 to 63·0)	8·7% (−48·9 to 76·9)	4·7% (−23·4 to 30·2)	50·0% (42·2 to 59·5)	−1·6% (−50·3 to 45·1)	0·1% (−22·0 to 16·2)
	65–69	48·1% (39·3 to 58·2)	−9·8% (−55·5 to 29·9)	2·0% (−16·8 to 14·1)	58·7% (50·4 to 68·3)	10·4% (−46·6 to 81·4)	9·5% (−18·1 to 34·1)	53·0% (45·3 to 62·4)	2·8% (−47·6 to 47·9)	6·2% (−16·5 to 21·4)
	70–74	44·7% (37·0 to 54·3)	0·6% (−53·9 to 45·1)	9·1% (−16·1 to 24·1)	65·1% (57·7 to 73·7)	19·8% (−42·9 to 90·3)	18·3% (−14·2 to 46·3)	52·4% (44·9 to 60·7)	12·8% (−44·9 to 61·8)	14·6% (−12·8 to 32·3)
	75–79	60·1% (49·9 to 72·1)	4·9% (−51·9 to 48·5)	13·5% (−17·3 to 31·5)	95·1% (80·7 to 111·2)	24·9% (−37·8 to 95·7)	25·0% (−10·4 to 56·2)	69·5% (58·8 to 81·7)	15·9% (−41·9 to 62·9)	19·2% (−12·3 to 39·9)
	≥80	113·8% (95·6 to 132·4)	48·7% (−38·0 to 113·0)	42·8% (−6·8 to 73·3)	174·1% (147·8 to 200·3)	57·6% (−26·8 to 141·2)	50·9% (−2·4 to 96·3)	123·8% (105·4 to 141·9)	51·5% (−29·5 to 115·0)	45·0% (−2·6 to 76·2)
Total		77·0% (63·3 to 91·8)	57·9% (−32·8 to 120·1)	26·7% (−6·9 to 46·0)	82·9% (67·4 to 100)	63·0% (−22·8 to 144·5)	26·9% (−8·7 to 56·2)	79·2% (65·9 to 93·7)	59·2% (−24·7 to 116·4)	26·6% (−5·3 to 46·8)

Data in parentheses are 95% uncertainty intervals. DALY=disability-adjusted life-year.

Both males and females had large percentage change increases in the incidence rate of falls between 1990 and 2019 (77·0% [95% UI 63·3–91·8] for females and 82·9% [67·4 to 100] for males]). Over the past three decades, the percentage change in incidence rate varied from 50·0% (42·1–59·5) for people aged 60–64 years to 123·8% (105·4–141·9) for people aged 80 years and older (appendix pp 12–14). Although the percentage change in the incidence rate of falls in the age 80 years and older group was significantly higher than in the other age groups, no significant differences were observed between the age groups for percentage change in mortality and DALY rates (table 2). In each specific age-sex group, a significant difference in percentage change was found between the age 80 years and older group and the other age groups in the incidence rate but not for mortality or DALY rates (table 2). The percentage change of the incidence rate among the age 70–74 years, 75–79 years, and 80 years and older groups was significantly higher for males than for females in the same age group, but similar trends were not seen for mortality and DALY rates (table 2).

All provinces had significant increases in the incidence rate of falls between 1990 and 2019, with the greatest percentage change being in Sichuan (148·5% [95% UI 125·5 to 171·4), followed by Yunnan (128·1% [109·1 to 147·8]) and Hubei (123·3% [105·8 to 144·5]), while the smallest percentage changes were in Jilin (14·7% [3·6 to 26·1]), followed by Heilongjiang (17·6% [6·6 to 29·7]) and Tibet (18·1% [8·7 to 29·0]; appendix pp 10–11). We found no significant changes in mortality rate across 31 provinces between 1990 and 2019 (appendix pp 10–14, 18). Between 1990 and 2019, the DALY rate decreased significantly in Jilin (–20·4% [–37·1 to –11·1]) and Heilongjiang (–15·3% [–29·4 to –5·3]), and increased significantly in six provinces (Sichuan, Yunnan, Hubei, Guizhou, Jiangxi, and Shanghai; appendix pp 10–14, 19). No significant change in DALY rate was found in other provinces (appendix pp 10–14, 20). We also found no significant differences in incidence, mortality, and DALY rates between male and female older people in each province between 1990 and 2019 (appendix pp 10–11). People aged 80 years and older had significantly greater increases in the percentage change of incidence rate of falls than those aged 70–74 years in all provinces, and then those aged 60–64, 65–69, and 75–79 years, in most provinces (appendix pp 12–14). We found no significant percentage changes in mortality and DALY rates between the age groups in all provinces (appendix pp 12–14).

## Discussion

With the data derived from GBD 2019, we here systematically described the incidence, mortality, and DALY rates of falls among people aged 60 years and older in mainland China in 2019, and presented the percentage change in the burden of falls between 1990 and 2019 at both national and subnational levels. This analysis allows comparisons of health loss because of falls over time across age groups, sexes, and provinces. Our study had four key findings: (1) a substantial increase in the incidence rate of falls occurred in older people in mainland China between 1990 and 2019, regardless of sex, age, and province; (2) we found minor disparities in the incidence, mortality, and DALY rates of falls in 2019 between males and females; (3) people aged 80 years and older are at generally higher risk of falls than those aged 60–79 years; and (4) there was a large variation in the incidence and DALY rates of falls across 31 provinces between 1990 and 2019.

The substantial increase in the incidence rate of falls among people aged 60 years and older in mainland China is not surprising; it is consistent with the increasing burden of falls at a global level.^1^ There are two potential primary reasons for the increase we observed in this Chinese population. First, the proportion of people aged 65 years and older has been continuously increasing, from 5·6% in 1990 to 11·5% in 2019, because of increasing life expectancy in China.^21^, ^22^ The risk factors for falls differ in older people across the age range.^1^, ^23^, ^24^ The risk factors for falls among older people who are younger than 70 years could be mainly due to extrinsic factors, such as inappropriate footwear, insufficient lighting, and slippery floors. With increasing age, the oldest-old people (ie, aged ≥80 years) have falls primarily because of decline in their intrinsic capacity and functional ability as a consequence of somatic and psychological health issues, such as sarcopenia, osteoporosis, sleeping disturbance, multimorbidity, and frailty^1^, ^23^, ^25^, ^26^—all of which are risk factors of falls.^1^, ^24^, ^27^ As people age, they are more likely to be exposed to increasing numbers of risk factors and more severe health conditions, and therefore are at increased risk of falls.^1^ Second, although prevention of falls has been included in the Chinese National Essential Public Health Service Package since 2009, no specific interventions have been provided for older people in the primary health-care setting, except for general health advice.^28^ Among the existing programmes for prevention of falls in mainland China, there remains an absence of high-quality evidence to guide the scale-up of interventions to prevent falls or integration with health systems.^29^ Additionally, the conventional perception of falls as an inevitable age-related event that cannot be prevented is also prevalent among community-dwelling older people. The low self-awareness of fall-prevention measures could further hinder implementation of falls prevention in a timely and effective manner.^30^ Other factors that might also be associated with the burden of falls in mainland China include the shrinking and simplification of family size and structure, poor supportive care services, and more sedentary lifestyles than previously.^1^, ^31^ As a result, older people are more prone to have fall events than in previous decades. However, we found that the mortality rate remained relatively stable over the past three decades, despite the increased number of new falls, which is consistent with findings from other studies in mainland China of people aged 65 years and older.^5^ The increased incidence rate but stable mortality rate of falls among older people could be partly attributed to progress in the primary health-care system and emergency medicine in China over the study period.^32^, ^33^

Sex has been widely reported to be a risk factor for falls,^1^, ^8^ but we only identified significant disparities in the burden of falls between males and females in two domains. First, we identified a higher mortality rate in males aged 60–64 years than in females in the same age group in 2019. Second, we found a greater increase in incidence rate between 1990 and 2019 in males aged 70 years and older than in females in the same age group. This sex disparity in the burden of falls among older people was consistent with the WHO global health estimates for 2019.^1^ Despite minor sex disparities in the burden of falls identified in specific age-sex groups, overall we found that falls have become a common health issue for all older people in mainland China. However, sex-equitable implementation of programmes for the prevention of falls among older people should still be encouraged. Our findings also confirm previous research that the oldest-old age group (ie, aged ≥80 years) is the age group at the highest risk of falls. GBD 2019 found that falls were the leading cause of disability due to a fractured bone in 2019.^34^ Here, we found a substantial increase in the incidence rate of falls among the oldest-old age group over the past three decades in mainland China. Therefore, we might expect a substantial increase in bone fractures among older people in China, including hip fracture, which is one of the most costly injuries in China.^34^, ^35^, ^36^ Development of programmes for the prevention of falls could be a potentially cost-effective strategy for older people, particularly for the oldest-old age group, to reduce the increasing burden of bone fractures in mainland China.^34^, ^37^

Although we found no substantial geographical variation in the mortality rate for falls across 31 provinces in mainland China, the incidence and DALY rates differed in some provinces. In 2019, Zhejiang and Fujian had some of the highest incidence and DALY rates of falls. Sichuan, Yunnan, and Hubei had the largest increases in incidence and DALYs rates for falls over the past three decades. There is an absence of reliable evidence on the effectiveness of fall-prevention interventions for older people in China.^29^ Evidence-based programmes for the prevention of falls should be implemented and assessed in large, rigorously designed, population-based studies in these provinces most affected by falls, to generate robust evidence of strategies to aid prevention of falls among older people, which could be scaled up to other provinces and the entire country. Some of the lowest incidence and DALY rates of falls were observed in Jilin and Heilongjiang, which also had some of the smallest increases in the incidence rate of falls and had significant decreases in the DALY rate of falls between 1990 and 2019. However, we did not identify any intervention studies for fall prevention for older people in these two provinces during the past three decades.^29^ The reason why these two provinces have the lowest burden of falls among older people remains unclear. The variation in the burden of falls at the province level reflects regional inequality in mainland China. Given the importance of government financial engagement in fall prevention^1^ and the fiscal capacity of subnational governments in mainland China,^38^ national-level policies are needed to support the lower-income provinces to better prevent falls among older people—eg, making the built environment and public spaces more suitable for older people.

Despite a GBD 2017 report highlighting the burden of different injuries at the national and subnational levels and the change in burden over time in China, detailed information on specific injury types within different populations in 31 provinces was not provided.^3^ To our knowledge, this is the first study to systematically and comprehensively describe the burden of falls in terms of incidence, mortality, and DALY rates among people aged 60 years and older, by sex, across 31 provinces in mainland China in 2019, and to examine the trends in key outcome indicators over the past three decades. Our study provides three novel insights. First, we highlighted a substantial increase of the incidence rate of falls among older people in mainland China regardless of sex, age group, or geographical location. Additionally, we identified the most vulnerable age–sex groups and regions, which could inform policy to target future interventions. Second, we highlighted the urgent need for population-level efforts into fall prevention to halt the upward trend in the incidence of falls among older people in mainland China. This finding echoed the conclusion drawn from the GBD 2019 global study on bone fractures.^34^ Our study also identified a similar pattern to that identified in a study from Hong Kong that showed a significant increase in fall-related admission to hospital in both older and younger populations since 2005.^39^ Finally, despite the fact that we examined the burden of falls in older people in mainland China, our results could provide insight for other countries or regions to understand the burden of falls among older people because falls are now a common health challenge within the global context of ageing.

As part of the GBD 2019 study, our study shares the same limitations, which are widely described in published literature.^11^, ^12^, ^13^, ^34^ However, our study has several other limitations. First, the scarcity of reliable non-fatal injury data in China has been recognised in previous studies. Therefore, the non-fatal burden of falls among older people could be sensitive to the quantity and quality of raw data involved in the estimation process. More reliable data sources from China contributed to GBD 2019 than had done for previous iterations, which should alleviate this concern to a large extent but might introduce some detection bias in longitudinal analyses. Second, the Chinese National Mortality Surveillance System, which was our main data source to estimate the burden of fatal falls, has undergone several structural changes since 1978, with substantial increases in the number of surveillance points and population coverage around 2004 and 2013. These structural changes might be the cause of the sudden shifts in the mortality rate for falls observed between 2000 and 2010 in several provinces.^40^ Third, there was a paucity of high-quality, injury-related data sources in some provinces, particularly in underdeveloped regions. Despite the merits from the systematic and comprehensive methodology framework adopted in GBD, we still could not completely avoid the negative effects of data quality issues on the reliability of estimations of the fall burden in these provinces. Fourth, data for those aged 60 years and older in this study are not age standardised. The change in the burden of falls could be affected by the shift in age structure of the population. Population ageing is a major factor of the change in the burden of falls,^1^, ^23^, ^24^ and the incorporation of the ageing demographic transition could reflect the trend of the burden of falls in reality. Finally, we aimed to present comprehensive ecological analyses of the burden of falls in mainland China, nationally and subnationally, but determining the definite causal inferences of the change and disparity in the burden of falls among different populations and provinces was outside of the scope of this study.

In summary, although the overall mortality and DALY rates of falls have not significantly changed for older people in mainland China since 1990, the large increase in the incidence rate of falls highlights the importance of falls as a serious and increasing health-care problem and reinforces the urgent need for evidence-based, gender-equitable interventions for the prevention of falls for older people in mainland China. The estimates and trends from this study also highlight the priority age groups and provinces for these interventions. Additional efforts and resources from national and local governments are needed to create solid support to aid prevention and reduce the burden of falls as population ageing progresses in mainland China in the future. The examination of levels, trends, and potential drivers of the burden of falls in mainland China, and the differences in this burden among its provinces over the past three decades, could also provide much needed insight into the transition of disease burden due to injuries in low-income and middle-income countries for years to come.

## Data sharing

Some of these data presented here are publicly available on the Global Health Data Exchange website and additional data can be requested from IHME (please contact Haidong Wang; haidong@uw.edu).

## Declaration of interests

We declare no competing interests.

## References

[1] WHO (2021).

[2] Vieira ER, Palmer RC, Chaves PH (2016). Prevention of falls in older people living in the community. BMJ.

[3] Leilei D, Pengpeng Y, Haagsma JA (2019). The burden of injury in China, 1990-2017: findings from the Global Burden of Disease Study 2017. Lancet Public Health.

[4] Peng K, Tian M, Andersen M (2019). Incidence, risk factors and economic burden of fall-related injuries in older Chinese people: a systematic review. Inj Prev.

[5] Cheng P, Wang L, Ning P (2019). Unintentional falls mortality in China, 2006-2016. J Glob Health.

[6] Rao WW, Zong QQ, Lok GKI (2018). Prevalence of falls in adult and older adult psychiatric patients in China: a systematic review and comprehensive meta-analysis of observational studies. Psychiatry Res.

[7] Wu H, Ouyang P (2017). Fall prevalence, time trend and its related risk factors among elderly people in China. Arch Gerontol Geriatr.

[8] Kwan MM, Close JC, Wong AK, Lord SR (2011). Falls incidence, risk factors, and consequences in Chinese older people: a systematic review. J Am Geriatr Soc.

[9] Hu G, Rao K, Baker SP (2010). Non-fatal injuries among Chinese aged 65 years and older: findings from the Fourth National Health Services Survey. Inj Prev.

[10] Pengpeng Y, Ye J, Yuliang E (2021). A scoping review of national policies for healthy ageing in mainland China from 2016 to 2020. Lancet Reg Health West Pac.

[11] GBD 2019 Diseases and Injuries Collaborators (2020). Global burden of 369 diseases and injuries in 204 countries and territories, 1990–2019: a systematic analysis for the Global Burden of Disease Study 2019. Lancet.

[12] GBD 2019 Demographics Collaborators (2020). Global age-sex-specific fertility, mortality, healthy life expectancy (HALE), and population estimates in 204 countries and territories, 1950–2019: a comprehensive demographic analysis for the Global Burden of Disease Study 2019. Lancet.

[13] GBD 2019 Risk Factors Collaborators (2020). Global burden of 87 risk factors in 204 countries and territories, 1990–2019: a systematic analysis for the Global Burden of Disease Study 2019. Lancet.

[14] National Bureau of Statistics of China The classification of economic regions in mainland China. http://www.stats.gov.cn/ztjc/zthd/sjtjr/dejtjkfr/tjkp/201106/t20110613_71947.htm.

[15] James SL, Castle CD, Dingels ZV (2020). Estimating global injuries morbidity and mortality: methods and data used in the Global Burden of Disease 2017 study. Inj Prev.

[16] James SL, Lucchesi LR, Bisignano C (2020). The global burden of falls: global, regional and national estimates of morbidity and mortality from the Global Burden of Disease Study 2017. Inj Prev.

[17] Stevens GA, Alkema L, Black RE (2016). Guidelines for accurate and transparent health estimates reporting: the GATHER statement. Lancet.

[18] The National People's Congress of the People's Republic of China Law of the People's Republic of China on Protection of the rights and interests of the elderly. http://www.npc.gov.cn/npc/c30834/201901/47231a5b9cf94527a4a995bd5ae827f0.shtml.

[19] Institute for Health Metrics and Evaluation Falls — Level 3 cause. http://www.healthdata.org/results/gbd_summaries/2019/falls-level-3-cause.

[20] Institute for Health Metrics and Evaluation Global Burden of Disease Study 2019 (GBD 2019) data input sources tool. http://ghdx.healthdata.org/gbd-2019/data-input-sources?components=2&locations=6.

[21] World Bank Life expectancy at birth, total (years) - China. https://data.worldbank.org/indicator/SP.DYN.LE00.IN?locations=CN.

[22] World Bank Population ages 65 and above (% of total population) – China. https://data.worldbank.org/indicator/SP.POP.65UP.TO.ZS?locations=CN.

[23] WHO (2015).

[24] WHO (2007).

[25] Fan J, Yu C, Guo Y (2020). Frailty index and all-cause and cause-specific mortality in Chinese adults: a prospective cohort study. Lancet Public Health.

[26] Zhang Y, Zhou L, Liu S (2020). Prevalence, correlates and outcomes of multimorbidity among the middle-aged and elderly: findings from the China Health and Retirement Longitudinal Study. Arch Gerontol Geriatr.

[27] Essien SK, Feng CX, Sun W, Farag M, Li L, Gao Y (2018). Sleep duration and sleep disturbances in association with falls among the middle-aged and older adults in China: a population-based nationwide study. BMC Geriatr.

[28] Ministry of Health of China National essential public health service package specification (2009 edition). http://www.nhc.gov.cn/jws/s3581r/200910/fe1cdd87dcfa4622abca696c712d77e8.shtml?from=singlemessage.

[29] Ye P, Liu Y, Zhang J (2020). Falls prevention interventions for community-dwelling older people living in mainland China: a narrative systematic review. BMC Health Serv Res.

[30] Hongman W (2020).

[31] WHO (2015).

[32] Li X, Lu J, Hu S (2017). The primary health-care system in China. Lancet.

[33] Shi X, Bao J, Zhang H (2020). Emergency medicine in China: a review of the history of progress and current and future challenges after 40 years of reform. Am J Emerg Med.

[34] GBD 2019 Fracture Collaborators (2021). Global, regional, and national burden of bone fractures in 204 countries and territories, 1990–2019: a systematic analysis from the Global Burden of Disease Study 2019. Lancet Healthy Longev.

[35] Zhang C, Feng J, Wang S (2020). Incidence of and trends in hip fracture among adults in urban China: a nationwide retrospective cohort study. PLoS Med.

[36] Wang Y, Cui H, Zhang D, Zhang P (2018). Hospitalisation cost analysis on hip fracture in China: a multicentre study among 73 tertiary hospitals. BMJ Open.

[37] Zhang LL, Dalal K, Yin MM, Yuan DG, Andrews JY, Wang SM (2012). The KAP evaluation of intervention on fall-induced injuries among elders in a safe community in Shanghai, China. PLoS One.

[38] National Bureau of Statistics of China (2020). China statistical yearbook. http://www.stats.gov.cn/tjsj/ndsj/2020/indexch.htm.

[39] Tang CTL, Sing CW, Kwok TCY, Li GHY, Cheung CL (2021). Secular trends in fall-related hospitalizations in adolescents, youth and adults: a population-based study. Lancet Reg Health West Pac.

[40] Liu S, Wu X, Lopez AD (2016). An integrated national mortality surveillance system for death registration and mortality surveillance, China. Bull World Health Organ.

