# Supplementary material for: Burden of falls among people aged 60 years and older in mainland China, 1990–2019: findings from the Global Burden of Disease Study 2019
Source: Lancet Public Health. 2021 Nov 25;6(12):e907–18. doi: 10.1016/S2468-2667(21)00231-0 (PMC8646839; doi:10.1016/S2468-2667(21)00231-0)
Supplement: Supplementary appendix [file mmc1.pdf]

THE LANCET
Public Health

Supplementary appendix

This appendix formed part of the original submission and has been peer reviewed. We post it as supplied by the authors.

Supplement to: Ye P, Er Y, Wang H, et al. Burden of falls among people aged 60 years and older in mainland China, 1990–2019: findings from the Global Burden of Disease Study 2019. *Lancet Public Health* 2021; **6:** e907–18.

Appendix

We searched for publications reporting the epidemiology of falls among older people in mainland China through Ovid Medline and Embase library until July 1, 2020 from database inception, with language restricted to English and Chinese. We included reviews or original studies which reported the epidemiological status, including incidence, prevalence, mortality, and disability-adjusted life years (DALY), of falls in people aged 60 years and older at the national level or subnational level without any restriction on physical and physiological conditions. Irrelevant studies were excluded by screening the titles and abstracts. There were 172 studies yielded, while 41 studies were identified as duplicates. The full text of remaining studies were further reviewed, of which 16 studies met the study criteria.

Table 1: Search terms used in Ovid Embase to assess the state of existing evidence

No.	Search terms	Results
1	exp falling/	43335
2	exp global disease burden/	3605
3	exp mortality/ or exp all cause mortality/ or exp premature mortality/ or exp epidemiological data/ or exp epidemiology/	1128677
4	exp incidence/ or exp epidemiological data/ or exp epidemiology/	692077
5	exp prevalence/ or exp epidemiological data/ or exp epidemiology/	1042519
6	exp disability-adjusted life year/ or exp disease burden/	27645
7	2 or 3 or 4 or 5 or 6	2217405
8	China/	239383
9	Chinese/	66617
10	8 or 9	299125
11	exp aged/ or exp frail elderly/ or exp very elderly/	3357584
12	1 and 7 and 10 and 11	109

Table 2: GATHER Checklist

Item #	Checklist item Reported on	Reported on page #
**Objectives and funding**		
1	Define the indicator(s), populations (including age, sex, and geographic entities), and time period(s) for which estimates were made.	Main Pg. 5-7
2	List the funding sources for the work.	Main Pg. 9
**Data Inputs**		
For all data inputs from multiple sources that are synthesised as part of the study:		
3	Describe how the data were identified and how the data were accessed.	Main Pg. 5-7
4	Specify the inclusion and exclusion criteria. Identify all ad-hoc exclusions.	Main Pg. 6
5	Provide information on all included data sources and their main characteristics. For each data source used, report reference information or contact name/institution, population represented, data collection method, year(s) of data collection, sex and age range, diagnostic criteria or measurement method, and sample size, as relevant.	Main Pg.9<br>Appendix (p 2)<br>http://ghdx.healthdata.org/gbd-2019/datainput-sources
6	Identify and describe any categories of input data that have potentially important biases (e.g., based on characteristics listed in item 5).	http://ghdx.healthdata.org/gbd-2019/datainput-sources
For data inputs that contribute to the analysis but were not synthesised as part of the study:		
7	Describe and give sources for any other data inputs.	N/A
For all data inputs:		
8	Provide all data inputs in a file format from which data can be efficiently extracted (e.g., a spreadsheet rather than a PDF), including all relevant metadata listed in item 5. For any data inputs that cannot be shared because of ethical or legal reasons, such as third-party ownership, provide a contact name or the name of the institution that retains the right to the data.	http://ghdx.healthdata.org/gbd-2019/datainput-sources
**Data analysis**		
9	Provide a conceptual overview of the data analysis method. A diagram may be helpful.	Main Pg. 6-9
10	Provide a detailed description of all steps of the analysis, including mathematical formulae. This description should cover, as relevant, data cleaning, data pre-processing, data adjustments and weighting of data sources, and mathematical or statistical model(s).	Main Pg. 6-9
11	Describe how candidate models were evaluated and how the final model(s) were selected.	N/A
12	Provide the results of an evaluation of model performance, if done, as well as the results of any relevant sensitivity analysis.	N/A
13	Describe methods for calculating uncertainty of the estimates. State which sources of uncertainty were, and were not, accounted for in the uncertainty analysis.	Main Pg. 8-9
14	State how analytic or statistical source code used to generate estimates can be accessed.	https://github.com/ihmeuw/ihme-modeling/tree/main/gbd_2019
**Results and Discussion**		
15	Provide published estimates in a file format from which data can be efficiently extracted.	http://ghdx.healthdata.org/gbd-results-tool
16	Report a quantitative measure of the uncertainty of the estimates (e.g. uncertainty intervals).	Main Pg. 10-12, tables and appendix (4-14)
17	Interpret results in light of existing evidence. If updating a previous set of estimates, describe the reasons for changes in estimates.	Main Pg. 3-4
18	Discuss limitations of the estimates. Include a discussion of any modelling assumptions or data limitations that affect interpretation of the estimates.	Main Pg. 16

Table 3: Data sources closely related to the estimation of falls burden in China in GBD 2019

Type	Name
Administrative data	• China - Shanghai Hospital Data<br>• China National Injury Surveillance System Bed Capacity by Hospital and Province<br>• Euromonitor Passport - Alcoholic Drinks Statistics
Census	• China Population and Housing Census<br>• China Census of population / China National Population Census<br>• China Sample Population Census
Demographic surveillance	• China Population Estimates
Disease registry	• China National Injury Surveillance System
Environmental monitoring	• China National Environmental Monitoring Center Network
Epi surveillance	• China Chronic Disease and Risk Factor Surveillance
Financial record	• China National Data GDP by Province<br>• China Gross Domestic Product by Province
Report	• China Statistical Yearbook<br>• China Health Statistics Yearbook<br>• China Health and Family Planning Statistics Yearbook<br>• China Environmental Quality Report<br>• China Population Statistics Yearbook<br>• China Demographic Yearbook<br>• China Energy Statistical Yearbook<br>• China - Review of the 100 County Growth Survey Procedures and Results<br>• Adult Mortality in China: Levels, Patterns, and Causes
Modeled data	• IHME GBD China Cause-Specific Mortality Estimates
Survey	• China World Poll<br>• Chinese Family Panel Studies Follow-Up<br>• China Health and Retirement Longitudinal Study<br>• China Comprehensive Social Survey Education Data<br>• China Sample Survey on Population Changes<br>• China Injury Comprehensive Surveillance Study<br>• China National Health Services Survey<br>• China Longitudinal Survey on Rural Urban Migration<br>• China WHO Study on Global AGEing and Adult Health<br>• China Health and Nutrition Survey<br>• China Zhuhai Study 2006-2007<br>• China National Cause of Death Sample Survey<br>• China National Nutrition Survey<br>• China Prospective Urban and Rural Epidemiological Study<br>• China World Health Survey<br>• China Household Income Project<br>• China WHO Multi-country Survey Study on Health and Health System Responsiveness<br>• China Health and Family Life Survey<br>• China Longitudinal Healthy Longevity Survey
Vital registration	• China Disease Surveillance Points<br>• China Vital Registration - Death Counts by Cause, County, Age, and Daily Temperature<br>• China Mortality Registration and Reporting System<br>• China Disease Surveillance Points and Information System for Disease Control and Prevention Deaths

Note: 1. The details of data sources above and others used to estimate the disease burden in China are listed in the website of Global Health Data Exchange (http://ghdx.healthdata.org/gbd-2019/data-input-sources?components=2&locations=6). 2. There were also relevant peer-reviewed articles involved in the estimation of falls burden in GBD.

Table 4: The incidence, mortality and DALY rates of falls among older people aged 60 years and older by sex in mainland China and 31 provinces in 2019

	Female			Male			Total		
Province	Incidence rate	Mortality rate	DALY rate	Incidence rate	Mortality rate	DALY rate	Incidence rate	Mortality rate	DALY rate
Mainland China	4264·9 (3474·5 to 5166·7)	39·1 (18·9 to 50·5)	1172·9 (854·5 to 1481)	3294·6 2618 to 4064·9)	39·3 (21·9 to 52·6)	1310·6 (937·6 to 1649·3)	3799·4 (3062·4 to 4645)	39·2 (21·8 to 48·8)	1238·9 (920·5 to 1553·2)
Anhui	3321·1 (2639·4 to 4099·1)	30·4 (18·6 to 41·8)	911·9 (703·3 to 1149·2)	2603·5 (2062·3 to 3272·3)	32·4 (22·2 to 42·1)	1084·4 (832·4 to 1344·9)	2972·4 (2390·5 to 3663·7)	31·3 (21·7 to 39·9)	995·7 (777·0 to 1234·5)
Beijing	6294 (5164·6 to 7711·3)	33·8 (15·8 to 47·8)	1377·7 (1042·3 to 1783·9)	4251·6 (3413 to 5262·3)	29·9 (17·0 to 42·0)	1205·0 (910·8 to 1540)	5323·0 (4357·1 to 6486·2)	31·9 (17·8 to 41·3)	1295·6 (988·6 to 1667·9)
Chongqing	4371·3 (3531·9 to 5388·1)	46·8 (21·8 to 63·8)	1367·4 (948·2 to 1759·6)	3815·3 (2996·1 to 4790·2)	56·3 (23·6 to 83·9)	1714 (1086·3 to 2293·6)	4101·3 (3281·8 to 5053·6)	51·4 (24·8 to 70·5)	1535·7 (1052·4 to 1966·9)
Fujian	7409·1 (6026·4 to 8959·5)	121·3 (19·1 to 181·1)	2463·4 (1214·1 to 3236·8)	4483·6 (3548·5 to 5432·1)	80·6 (19·4 to 132·8)	2014·5 (1050·1 to 2811·2)	5990·7 (4857·6 to 7249·2)	101·6 (20·9 to 145·3)	2245·7 (1158·6 to 2918)
Gansu	2085·1 (1672 to 2589·7)	18·7 (14·2 to 28·3)	643·7 (513 to 808·7)	1862·6 (1456·1 to 2369·5)	27·1 (19·9 to 39·6)	896 (708·4 to 1133·3)	1976·3 (1564·7 to 2451·5)	22·8 (18·2 to 30·9)	767 (618·8 to 936·7)
Guangdon g	6114·8 (4941·4 to 7393)	45·8 (17·7 to 67·7)	1482·6 (1064·5 to 1922·7)	3940·8 (3159·3 to 4871)	38·1 (18·4 to 54·6)	1342·2 (953·0 to 1727·0)	5061·7 (4093·9 to 6170·9)	42·0 (19·8 to 56·9)	1414·6 (1021·2 to 1796·6)
Guangxi	4125·0 (3224·8 to 5189·6)	54·0 (21·4 to 74·6)	1338·9 (858·9 to 1710·5)	3274·4 (2527·7 to 4149·6)	53·3 (23·2 to 78·9)	1493·4 (931·8 to 2014)	3727·4 (2902·3 to 4694·9)	53·7 (25·4 to 73·9)	1411·1 (942·1 to 1801·8)
Guizhou	4677·4 (3758·3 to 5734·4)	52·6 (23·4 to 78·2)	1601·9 (1092·4 to 2057·4)	3729·1 (2978·7 to 4667·6)	63·2 (25·2 to 94·8)	1896·0 (1139·8 to 2550)	4228·8 (3430·2 to 5178·3)	57·6 (26·2 to 81·9)	1741·0 (1155·6 to 2228·6)
Hainan	4405·1 (3594·0 to 5396·9)	41·6 (23·0 to 57·0)	1190 (893·2 to 1498·6)	3368·1 (2717·3 to 4130·2)	31·2 (20·0 to 42·8)	1126·9 (860·4 to 1410·0)	3893·4 (3188 to 4749)	36·5 (24·1 to 46·2)	1158·9 (901·7 to 1433·2)
Hebei	2193·4 (1778·1 to 2715·2)	8·6 (4·8 to 23·8)	515·8 (379·4 to 729·9)	2147·5 (1680·7 to 2709)	16·8 (9·6 to 33·9)	781·1 (560·3 to 1084·8)	2171·6 (1740 to 2706·8)	12·5 (7·6 to 26·5)	642·1 (465·4 to 880·9)
Heilongjia ng	1939·1 (1533·0 to 2415·0)	9·4 (6·3 to 19·8)	478·3 (358·8 to 645·9)	2037·3 (1568·8 to 2594·4)	15·8 (9·2 to 32)	727·8 (520·8 to 1024·2)	1985·6 (1553·8 to 2482·4)	12·4 (8·0 to 24·2)	596·4 (440·4 to 802·3)
Henan	2654·1	18·5	689·1	2468·8	25·5	1018·0	2567·9	21·8	842·2

	(2063·9 to 3365·6)	(13·6 to 25·6)	(544·3 to 874)	(1850·1 to 3166·2)	(19·3 to 33·1)	(801·5 to 1258·6)	(1975·8 to 3271·6)	(17·4 to 27·4)	(679·8 to 1045·9)
Hubei	4672·4 (3678·9 to 5820·6)	49 (16·8 to 68·7)	1373 (896·7 to 1747·6)	3596 (2752·7 to 4504·5)	51·3 (20·4 to 77·9)	1535·3 (959·4 to 2071·6)	4151·2 (3242·4 to 5150·1)	50·1 (20·0 to 69·5)	1451·6 (946·3 to 1853·1)
Hunan	3344·8 (2678·8 to 4144·9)	30 (19·8 to 38·5)	965·0 (737·0 to 1201·0)	2791·4 (2190·5 to 3527·2)	37·3 (22·8 to 50·8)	1209 (865·7 to 1528·9)	3075·9 (2446·1 to 3791·7)	33·5 (23·2 to 42·6)	1083·5 (832·9 to 1343·0)
Inner Mongolia	2016·3 (1572·9 to 2539·7)	9·7 (6·4 to 21·7)	494·6 (373·3 to 675·5)	2000·9 (1526·3 to 2567·0)	16·8 (9·8 to 31·5)	744·3 (544·2 to 1034·3)	2008·8 (1546·4 to 2548·3)	13·1 (8·7 to 24·6)	616·1 (458·6 to 820·5)
Jiangsu	6328·3 (5180·1 to 7596·7)	61·9 (19·3 to 97·4)	1616 (1061·4 to 2089·2)	4332·3 (3456·4 to 5319·9)	46·6 (19·6 to 68·5)	1549·6 (1047·2 to 2029·2)	5377·4 (4378·1 to 6427·9)	54·6 (20·9 to 79·6)	1584·4 (1072·2 to 2013·1)
Jiangxi	5094 (4095·1 to 6279·5)	36·3 (20·1 to 48·0)	1346·8 (1020·4 to 1707·0)	4053·1 (3150·0 to 5189·6)	40·2 (19·6 to 58·9)	1537·5 (1061·9 to 1993·1)	4593·2 (3630·6 to 5742·2)	38·2 (21·9 to 49·2)	1438·5 (1067 to 1823)
Jilin	1897·6 (1464·2 to 2392·1)	8·9 (5·8 to 21·0)	464·9 (339·4 to 655·4)	1887·8 (1424·9 to 2414·4)	13·5 (6·9 to 28·8)	634·0 (442·9 to 897·9)	1892·9 (1449·5 to 2402·4)	11·1 (7·3 to 22·1)	547·0 (402·1 to 738·5)
Liaoning	2441·4 (1980·8 to 2968·6)	13·7 (9·5 to 21·1)	579·9 (444·3 to 748·9)	2190·2 (1654·7 to 2801·3)	20·2 (14·6 to 31·3)	827·1 (639·0 to 1051·7)	2322·3 (1832·4 to 2862·3)	16·8 (12·8 to 24·4)	697 (546·4 to 875·8)
Ningxia	3157·9 (2412·2 to 4028·3)	27·3 (16·2 to 36·3)	918·5 (695·9 to 1162·2)	2421·4 (1826·2 to 3103·6)	31·9 (22·1 to 41·4)	1055·2 (819·8 to 1301·8)	2800·3 (2142·4 to 3549)	29·6 (21·0 to 36·7)	984·9 (788·3 to 1210·8)
Qinghai	2484·9 (1920·1 to 3172·1)	26·6 (20·6 to 34·2)	922·4 (731·7 to 1128·5)	2196·2 (1671·8 to 2834·1)	36·6 (25·7 to 47·0)	1213·9 (954·8 to 1482)	2345·3 (1807·4 to 2981·8)	31·5 (25·4 to 37·7)	1063·3 (870·9 to 1277·3)
Shaanxi	3698·1 (2872·1 to 4685·4)	32·3 (15·9 to 44·2)	1080·0 (775 to 1382·6)	3086·8 (2358 to 3919)	42·0 (20·9 to 60·4)	1347·3 (900·9 to 1769·1)	3400·5 (2653·2 to 4300·5)	37·1 (20·1 to 49·2)	1210·1 (859·4 to 1543·8)
Shandong	2966·9 (2395 to 3629·3)	15·8 (11·4 to 24·9)	704·2 (541·5 to 903·2)	2545·7 (2014·0 to 3154·7)	21·8 (16·0 to 31·7)	918 (714·6 to 1148·6)	2768·7 (2230 to 3399·5)	18·6 (14·5 to 25·6)	804·8 (633·7 to 1007)
Shanghai	7419·1 (6133·6 to 8925·7)	58·4 (19·8 to 82·8)	1781·7 (1270·6 to 2296·2)	4636·6 (3711·3 to 5665·5)	35·2 (16·0 to 53·5)	1412·1 (1014·8 to 1822·5)	6062·5 (4981·0 to 7266·8)	47·1 (19·8 to 63·1)	1601·5 (1158·5 to 2057·5)
Shanxi	2672·1	16·7 (12 to 24·6)	664·6 (520·1 to 850·4)	2484·5	24·3 (17·6 to 32·5)	957·7 (752·7 to 1191·3)	2580·1	20·4 (15·7 to 26·6)	808·4 (642·5 to 1008·9)

	(2149·5 to 3280·5)			(1945·2 to 3116·8)			(2076·1 to 3191·4)		
Sichuan	5454·9 (4360·7 to 6659·9)	52·4 (19·5 to 71·0)	1647·5 (1080·8 to 2095·3)	4223·7 (3323·6 to 5302·8)	61·4 (23·5 to 93·6)	1931·1 (1159·4 to 2626)	4863·9 (3878·2 to 5951·6)	56·7 (23·9 to 78·5)	1783·6 (1154 to 2302·4)
Tianjin	3250·8 (2549·5 to 4063·5)	20·3 (14·0 to 28·0)	754·4 (590·4 to 956·6)	2838·6 (2162·1 to 3611·6)	23·5 (17·1 to 29·1)	945·5 (738·4 to 1164·4)	3048·6 (2374·1 to 3800·1)	21·9 (17·1 to 27·2)	848·2 (667·8 to 1052·3)
Tibet	2881·6 (2195·1 to 3653·2)	44·0 (24·6 to 62·0)	1325·7 (996·5 to 1680·3)	2312·9 (1729·4 to 2992·6)	52·5 (26·9 to 83·4)	1624·9 (1101·7 to 2211·4)	2618·4 (1990·3 to 3330·3)	48·0 (28·9 to 67·6)	1464·2 (1079·1 to 1848)
Xinjiang	2266·5 (1800·5 to 2828·5)	17·8 (12·8 to 30·7)	678·8 (535·5 to 883·4)	1805·6 (1420·8 to 2258·7)	25·4 (16·1 to 41)	877·5 (663·8 to 1154·7)	2032·4 (1608·4 to 2541·8)	21·7 (15·7 to 33·7)	779·7 (609·6 to 997·6)
Yunnan	5467·7 (4435·0 to 6660·1)	77·8 (24·0 to 104·1)	1984·6 (1162·4 to 2501·2)	4154·2 (3307·1 to 5160·5)	82·6 (27·3 to 126·4)	2257·7 (1212·6 to 3118·5)	4850·0 (3898·5 to 5930·1)	80·1 (27·9 to 108·2)	2113·1 (1221·5 to 2718)
Zhejiang	8425·6 (6863·9 to 10076·1)	108·5 (19·2 to 158·2)	2383·7 (1332·9 to 3082·9)	5859·4 (4745·5 to 7169·3)	77·4 (19·2 to 125·9)	2128·1 (1184·9 to 2910·3)	7170·9 (5835·3 to 8649·8)	93·3 (20·9 to 130·8)	2258·7 (1278·7 to 2932·1)

Data in parentheses are 95% uncertainty intervals. DALY=disability-adjusted life-year.

Table 5: The incidence, mortality and DALY rates of falls among older people aged 60 years and older by age groups in mainland China and 31 provinces in 2019

	60 to 64			65 to 69			70 to 74			75 to 79			80 plus			Total		
Province	Incidence rate	Mortality rate	DALY rate	Incidence rate	Mortality rate	DALY rate	Incidence rate	Mortality rate	DALY rate	Incidence rate	Mortality rate	DALY rate	Incidence rate	Mortality rate	DALY rate	Incidence rate	Mortality rate	DALY rate
Mainland China	2477·8 (1580 to 3514·7)	9·3 (5·5 to 12·2)	713·8 (539·0 to 900·2)	2741·8 (1822·2 to 3945·5)	12·5 (7·4 to 16·2)	848·2 (642·9 to 1077·2)	3298·6 (2151·9 to 4597·8)	22·5 (12·6 to 28·6)	1122 (827·9 to 1424)	4876·7 (3202 to 7195·2)	46·1 (25·6 to 57·8)	1590·2 (1172·3 to 2001·2)	9419·2 (7007·4 to 12418·4)	198·3 (104·2 to 249·5)	3349·5 (2356·8 to 4128·9)	3799·4 (3062·4 to 4645)	39·2 (21·8 to 48·8)	1238·9 (920·5 to 1553·2)
Anhui	1981 (1247·5 to 2822·3)	8·5 (5·1 to 12·2)	603·7 (452·4 to 776·4)	2131·4 (1371·4 to 3113·8)	10·1 (6·7 to 13·9)	678·5 (512·5 to 865·4)	2507·5 (1606·1 to 3551·5)	17·3 (11·3 to 23·6)	870·5 (663·8 to 1101·8)	3662·3 (2331 to 5453·1)	32·6 (22·9 to 43·4)	1178·4 (925·7 to 1482)	6708·8 (4910·1 to 9113·5)	143·2 (98·6 to 181·7)	2441·4 (1912 to 2959·5)	2972·4 (2390·5 to 3663·7)	31·3 (21·7 to 39·9)	995·7 (777 to 1234·5)
Beijing	3199·8 (2054 to 4642·8)	4·2 (3 to 6)	672·9 (505·6 to 877·9)	3719·6 (2514·6 to 5328·1)	6·1 (4·3 to 8·3)	842 (625·7 to 1102·7)	4304·8 (2803·4 to 6195·3)	12·0 (7·9 to 16·2)	1112·9 (825·3 to 1441·7)	6601·9 (4232·2 to 9674·9)	27·8 (15·9 to 37·5)	1593·1 (1198·6 to 2081·4)	13441·5 (10042·5 to 17909)	169·0 (86·1 to 226·3)	3572·7 (2616·3 to 4552·5)	5323 (4357·1 to 6486·2)	31·9 (17·8 to 41·3)	1295·6 (988·6 to 1667·9)
Chongqing	2823·2 (1818·5 to 3990·1)	14·2 (5·9 to 22)	929 (635·2 to 1237·6)	3068 (2006·3 to 4477·3)	19·7 (7·9 to 30·3)	1099·1 (747·5 to 1456·8)	3634·9 (2372·2 to 5117·9)	32·6 (14·2 to 48·6)	1398·4 (943·8 to 1844·9)	5177·8 (3313·5 to 7676·4)	66·0 (28·9 to 97·1)	1976·3 (1312·7 to 2590·4)	8836·4 (6401·3 to 11999·8)	220·8 (114·4 to 295·9)	3596·3 (2453 to 4534·2)	4101·3 (3281·8 to 5053·6)	51·4 (24·8 to 70·5)	1535·7 (1052·4 to 1966·9)
Fujian	3464·0 (2249·5 to 4847)	13·8 (4·5 to 20·9)	990·1 (652·3 to 1297·8)	4030·7 (2683·5 to 5736·6)	21·3 (6·0 to 32·0)	1251·4 (787·3 to 1628·9)	5011·8 (3344·8 to 6951·4)	45·7 (10·5 to 69·6)	1831·3 (1003·0 to 2412·0)	7763·6 (5065·8 to 11173·1)	106·2 (22·2 to 159·9)	2890 (1453·1 to 3932·2)	16053·4 (11867·7 to 21227·5)	553·6 (102·4 to 794·8)	7394·9 (3064·6 to 9865·2)	5990·7 (4857·6 to 7249·2)	101·6 (20·9 to 145·3)	2245·7 (1158·6 to 2918)
Gansu	1436·5 (898·9 to 2060·4)	7·2 (5·2 to 10·0)	501·9 (389·2 to 627·3)	1491·9 (952·9 to 2212·9)	9·0 (6·5 to 12·9)	564·3 (438·9 to 703)	1738·5 (1106·8 to 2482·6)	15·9 (12 to 22)	726·1 (574·1 to 888·8)	2525·5 (1596·4 to 3845·8)	29·6 (21·6 to 42·6)	972·9 (773·3 to 1218·1)	4460·6 (3190 to 6098·8)	106·2 (84·4 to 141·7)	1832·9 (1490·5 to 2264)	1976·3 (1564·7 to 2451·5)	22·8 (18·2 to 30·9)	767·0 (618·8 to 936·7)
Guangdong	3018·0 (1883·1 to 4314·7)	7·0 (4·0 to 9·9)	738·5 (554·5 to 954·1)	3442·6 (2246·9 to 5030·5)	10·0 (5·2 to 14·2)	901·3 (664 to 1183·3)	4203·9 (2720·9 to 6018·4)	19·0 (8·9 to 27·3)	1203·9 (869·3 to 1558·8)	6449·3 (4231·2 to 9500·3)	42·8 (18·8 to 61·9)	1757·3 (1235·2 to 2292·8)	12311·3 (9170·6 to 16174·2)	202·8 (89·1 to 277·3)	3777·7 (2558·7 to 4881·6)	5061·7 (4093·9 to 6170·9)	42·0 (19·8 to 56·9)	1414·6 (1021·2 to 1796·6)
Guangxi	2320·9 (1388 to 3479·8)	13·0 (6·5 to 20·2)	773·8 (536·9 to 1025·9)	2619·6 (1640·5 to 3929·5)	17·8 (8·3 to 26·8)	922·0 (631·6 to 1203·1)	3243·0 (1997 to 4721·6)	31·6 (14·2 to 47·0)	1240·7 (836·9 to 1646·1)	4874·7 (3056·3 to 7538·8)	65·2 (28·5 to 93·5)	1827·2 (1187·5 to 2386·4)	8722·3 (6191·2 to 12079·7)	239·6 (110·5 to 325·8)	3686·1 (2335·4 to 4679·5)	3727·4 (2902·3 to 4694·9)	53·7 (25·4 to 73·9)	1411·1 (942·1 to 1801·8)
Guizhou	3026·7 (1934·4 to 4275·9)	18·1 (7·1 to 29·5)	1080·3 (707·2 to 1451·1)	3313·9 (2237·2 to 4697·7)	24·6 (9·5 to 40·0)	1276·6 (844·7 to 1709·8)	3850·4 (2551·5 to 5400·8)	43·2 (16·3 to 67·3)	1687·9 (1072·9 to 2244·2)	5382·3 (3570·9 to 7812·8)	76·8 (33·0 to 117·2)	2256·2 (1479·8 to 3018·5)	8716·8 (6432·7 to 11497·6)	238·8 (121·3 to 340·4)	3986·4 (2673·4 to 5161·0)	4228·8 (3430·2 to 5178·3)	57·6 (26·2 to 81·9)	1741 (1155·6 to 2228·6)
Hainan	2393·3 (1537·6 to 3397·2)	7·6 (5·1 to 11)	619·7 (475·3 to 781·3)	2767·6 (1870·2 to 3884·6)	10·5 (7·0 to 14·6)	754·3 (583·7 to 962·8)	3418·4 (2219·8 to 4874·6)	20·1 (12·8 to 28·0)	1032·8 (788 to 1318·3)	5227·4 (3486·3 to 7511·4)	44·4 (26·9 to 61·1)	1539·5 (1163·7 to 1947·5)	9159 (6857·4 to 11992·7)	170·6 (109·7 to 219·1)	3079·5 (2346·1 to 3775·3)	3893·4 (3188 to 4749)	36·5 (24·1 to 46·2)	1158·9 (901·7 to 1433·2)
Hebei	1638·6 (1059·5 to 2340·3)	4·7 (2·6 to 9·2)	460·1 (331 to 620·4)	1751 (1152·5 to 2504·2)	5·7 (3·1 to 11·8)	521·4 (375·2 to 694·1)	2016·6 (1288·4 to 2868·4)	9·4 (5·0 to 20·0)	641·0 (462·1 to 855·3)	2923·9 (1883·8 to 4365·9)	18·2 (10·1 to 38·6)	848·9 (614·8 to 1183·9)	4909 (3576·7 to 6471·7)	61·8 (35·9 to 141·4)	1456·1 (1038·1 to 2257·2)	2171·6 (1740 to 2706·8)	12·5 (7·6 to 26·5)	642·1 (465·4 to 880·9)
Heilongjiang	1566·9	4·5	423·7	1667·4	5·7	487·3	1830·1	10·0	612·7	2558·9	19·1	808·7	4390	64·4	1392·6	1985·6	12·4	596·4

	(946·6 to 2266·6)	(2·5 to 9·1)	(310·6 to 573·4)	(1061·4 to 2405·6)	(3·2 to 11·6)	(352·5 to 644·7)	(1170·6 to 2583·7)	(5·8 to 19·6)	(449·3 to 810·6)	(1620·4 to 3910·5)	(11·6 to 37·7)	(593·0 to 1101·6)	(3145·6 to 5998·4)	(42·1 to 127·7)	(1025·7 to 2056·2)	(1553·8 to 2482·4)	(8·0 to 24·2)	(440·4 to 802·3)
Henan	1836·4 (1088·1 to 2783·5)	7·5 (4·9 to 10·6)	581·5 (444·2 to 741)	1913·5 (1164·1 to 2891·8)	8·5 (6·1 to 11·3)	636·5 (496·6 to 811·1)	2201·4 (1339·1 to 3219·9)	12·9 (9·3 to 17·7)	768·0 (599·8 to 971·6)	3232·1 (2011·6 to 5098·7)	24·9 (18·4 to 34·7)	1021·1 (809·6 to 1281·2)	6061·3 (4269 to 8454·1)	104·1 (83·7 to 133·4)	1978·8 (1612·2 to 2425·6)	2567·9 (1975·8 to 3271·6)	21·8 (17·4 to 27·4)	842·2 (679·8 to 1045·9)
Hubei	2708·9 (1714·9 to 4003·7)	12·4 (5·2 to 19·1)	836·4 (565·5 to 1111·7)	3050·3 (1916·0 to 4540·8)	16·8 (7·0 to 25·6)	992·1 (678·7 to 1296·1)	3722·7 (2345·2 to 5313·2)	30·3 (12·1 to 46·0)	1324·3 (872·0 to 1751·1)	5546·1 (3560·9 to 8441·3)	62·0 (25·2 to 90·8)	1909·4 (1225·9 to 2507·5)	10426·3 (7526·8 to 14206·8)	268·5 (102·9 to 369·3)	4147·2 (2472·0 to 5279·0)	4151·2 (3242·4 to 5150·1)	50·1 (20·0 to 69·5)	1451·6 (946·3 to 1853·1)
Hunan	2166·5 (1380·1 to 3055·5)	11·3 (6·0 to 16·5)	700·4 (491·8 to 904·1)	2350·8 (1533·7 to 3499)	13·8 (7·7 to 19·1)	791·9 (576·3 to 1020·6)	2763·6 (1802·9 to 3949·5)	23·7 (13·7 to 33·2)	1028·3 (745·7 to 1318·9)	3898·6 (2533·7 to 5818·3)	42·7 (27·0 to 56·9)	1377·8 (1040·8 to 1742·6)	6485 (4718·5 to 8804·8)	135·3 (102 to 165·5)	2423·2 (1930·8 to 2957·2)	3075·9 (2446·1 to 3791·7)	33·5 (23·2 to 42·6)	1083·5 (832·9 to 1343·0)
Inner Mongolia	1484·9 (924·8 to 2208)	4·7 (2·8 to 8·5)	437·5 (321·1 to 581·7)	1580·2 (986 to 2331·4)	5·7 (3·3 to 11·2)	492 (352·6 to 650·3)	1873 (1175·7 to 2716·9)	9·7 (6·0 to 18·5)	613·9 (449·6 to 818·9)	2746·6 (1699·2 to 4216·8)	19·1 (12·1 to 37·0)	823·7 (611·2 to 1123·5)	4725·1 (3410·7 to 6383·0)	66·8 (44·8 to 129·3)	1453·8 (1075·8 to 2140·5)	2008·8 (1546·4 to 2548·3)	13·1 (8·7 to 24·6)	616·1 (458·6 to 820·5)
Jiangsu	3216·3 (2055·6 to 4465·6)	8·6 (4·0 to 12·6)	799·7 (580·7 to 1045·6)	3558·2 (2408·2 to 4997·1)	11·8 (5·2 to 17·4)	967·0 (694·9 to 1255·4)	4228·2 (2777·1 to 5941·3)	21·0 (9·1 to 31·6)	1261·8 (895·2 to 1646)	6417·0 (4198·5 to 9415·5)	45·0 (19·2 to 67·2)	1806·9 (1260·0 to 2368·2)	13529·7 (10180·3 to 17752·8)	275·7 (97·8 to 408·0)	4460·7 (2682·4 to 5763·4)	5377·4 (4378·1 to 6427·9)	54·6 (20·9 to 79·6)	1584·4 (1072·2 to 2013·1)
Jiangxi	3360·9 (2153·1 to 4867·3)	11·7 (5·2 to 17·5)	915·0 (638·1 to 1189·3)	3629·9 (2357·7 to 5279·2)	14·6 (7·1 to 20·7)	1066·1 (769·9 to 1387·2)	4163 (2654·4 to 5934·6)	25·5 (12·8 to 35·1)	1379·6 (1000·7 to 1776·1)	5926·2 (3982·6 to 8680·1)	50·4 (26·1 to 68·4)	1900·0 (1387·3 to 2440·8)	9719·3 (7182·9 to 12816)	176·8 (109·9 to 225·2)	3434·5 (2600·9 to 4263·9)	4593·2 (3630·6 to 5742·2)	38·2 (21·9 to 49·2)	1438·5 (1067 to 1823)
Jilin	1371·3 (818·3 to 2073·5)	3·7 (2·1 to 7·5)	375·1 (267·1 to 507·2)	1499·0 (934·8 to 2236·8)	5.0 (3·0 to 9·9)	439·7 (316·4 to 579·9)	1773·4 (1097·4 to 2604·7)	8·3 (5·1 to 16·3)	549·3 (405·5 to 724·3)	2616·3 (1632·3 to 3979)	16·7 (10·7 to 31·2)	746·5 (542·5 to 999·1)	4441·4 (3137·2 to 6194·5)	55·8 (35·3 to 117·6)	1288·3 (931·7 to 1937)	1892·9 (1449·5 to 2402·4)	11·1 (7·3 to 22·1)	547·0 (402·1 to 738·5)
Liaoning	1652·4 (1028·5 to 2435·9)	5·5 (3·7 to 8·2)	471·0 (357·3 to 610·6)	1763·9 (1134 to 2555·8)	6·5 (4·3 to 10·3)	532·1 (401·8 to 672·4)	2055·5 (1322·7 to 2870·6)	10·9 (7·3 to 17·1)	666·7 (509·8 to 857·2)	3029·7 (1945 to 4470·5)	21·7 (14·9 to 34·2)	905·3 (694·6 to 1150·5)	5856·4 (4270·8 to 7965·7)	88·4 (66·4 to 127·3)	1762·1 (1410·3 to 2233·6)	2322·3 (1832·4 to 2862·3)	16·8 (12·8 to 24·4)	697 (546·4 to 875·8)
Ningxia	1915 (1142·9 to 2887·5)	9·3 (6·1 to 13·3)	630·0 (486·5 to 805·8)	2082·5 (1294·7 to 3139·5)	12·0 (8·1 to 16·7)	725·2 (568·1 to 912·5)	2582·3 (1621·9 to 3747·2)	20·7 (13·9 to 27·5)	938·2 (725·7 to 1175·8)	3865·6 (2457·7 to 5911·3)	43·3 (29·2 to 56·4)	1355·4 (1047·9 to 1676)	7017·9 (4939·1 to 9853·5)	150·5 (105·7 to 186)	2587·8 (2004·9 to 3146·3)	2800·3 (2142·4 to 3549)	29·6 (21·0 to 36·7)	984·9 (788·3 to 1210·8)
Qinghai	1741·1 (1051·1 to 2579·5)	11·8 (7·9 to 15·8)	741·0 (578·6 to 923·8)	1846·9 (1142·8 to 2767·7)	15·2 (10·7 to 20·5)	835·5 (650·9 to 1036·2)	2194·2 (1368·5 to 3212·7)	25·6 (18·7 to 33·5)	1054·6 (837·4 to 1284·5)	3144·4 (1959·1 to 4841)	46·5 (35·5 to 59)	1404·0 (1138·4 to 1723)	5176·1 (3613·4 to 7249·1)	140·7 (112 to 174·7)	2428·1 (2022·7 to 2893·7)	2345·3 (1807·4 to 2981·8)	31·5 (25·4 to 37·7)	1063·3 (870·9 to 1277·3)
Shaanxi	2387·2 (1469·4 to 3566)	12·1 (5·6 to 18·1)	776 (535·2 to 1027·9)	2648 (1655·7 to 3961·8)	15·8 (7·3 to 23·3)	903·1 (636·0 to 1186·2)	3163·4 (1964·9 to 4525·9)	26·7 (13·0 to 38·9)	1171·7 (817·4 to 1535)	4584·8 (2945·9 to 6927·7)	52·5 (26·2 to 72·9)	1642·1 (1155·4 to 2153·7)	7771·3 (5611·8 to 10686·2)	175·9 (100·5 to 227·5)	3019·3 (2150·1 to 3790)	3400·5 (2653·2 to 4300·5)	37·1 (20·1 to 49·2)	1210·1 (859·4 to 1543·8)
Shandong	1971·7	5·7 (4·2 to 7·8)	524·8 (402·7 to 668·0)	2100·0	6·9 (5·0 to 9·5)	602·7 (457·7 to 772·1)	2399·2	11·1 (8·1 to 16)	749·1 (578 to 953)	3446·6 (2233 to 5165·5)	21·5 (15·6 to 31·6)	999·9 (769·9 to 1270·8)	6208·9	86·2 (64·8 to 128·8)	1863·5	2768·7 (2230 to 3399·5)	18·6 (14·5 to 25·6)	804·8 (633·7 to 1007·0)

	(1257·6 to 2794·3)			(1380·7 to 3014·4)			(1558·9 to 3369·8)						(4559·7 to 8192·5)		(1469·5 to 2399·4)			
Shanghai	3509·5 (2267·6 to 4926·8)	5·7 (3 to 8)	811·5 (599·3 to 1061·2)	4109·9 (2757·1 to 5839·9)	8·5 (4·2 to 12·0)	1008 (737·5 to 1315·2)	4951 (3273 to 6993·6)	17·0 (8·0 to 24·0)	1342·1 (970·3 to 1745·4)	7528·3 (4864·6 to 11013·1)	36·8 (16·9 to 51·0)	1902·9 (1363 to 2491·9)	16527·8 (12386·7 to 21782)	268·1 (106·9 to 366·8)	4807·9 (3204·2 to 6095·4)	6062·5 (4981 to 7266·8)	47·1 (19·8 to 63·1)	1601·5 (1158·5 to 2057·5)
Shanxi	1926·7 (1242·2 to 2682)	6·9 (4·8 to 9·8)	549·5 (427·6 to 703·3)	2072·9 (1370·8 to 2974·1)	8·7 (6·1 to 12·0)	632·3 (491·8 to 811·8)	2330·1 (1506·3 to 3299·1)	14·0 (9·8 to 19·2)	783·8 (604·5 to 1002·5)	3305·2 (2150·1 to 4885·4)	26·1 (18·6 to 38·1)	1039·9 (808·9 to 1313)	5868·1 (4272·3 to 7819·8)	103·2 (81·1 to 135·7)	1973·9 (1597·8 to 2421·2)	2580·1 (2076·1 to 3191·4)	20·4 (15·7 to 26·6)	808·4 (642·5 to 1008·9)
Sichuan	3358 (2135·1 to 4839·8)	17·2 (6·6 to 26·9)	1107·3 (724·9 to 1483·5)	3663·2 (2445·2 to 5207·7)	23·5 (8·7 to 36·7)	1303 (850·5 to 1745)	4420·9 (2935·5 to 6158·6)	40·4 (15·5 to 60·8)	1693·4 (1078·5 to 2239)	6463·6 (4238·4 to 9357·8)	77·0 (30·9 to 113·4)	2343·5 (1493·1 to 3105·6)	10825·2 (8049·1 to 14201·6)	255·5 (111·7 to 349·9)	4295·1 (2741·8 to 5448·4)	4863·9 (3878·2 to 5951·6)	56·7 (23·9 to 78·5)	1783·6 (1154·0 to 2302·4)
Tianjin	1986·3 (1207·1 to 2956·5)	5·3 (3·7 to 7·1)	514·8 (397·8 to 654·5)	2200·6 (1365·1 to 3311·1)	6·5 (4·7 to 8·8)	597·5 (457·9 to 770·1)	2759·3 (1704·5 to 3949·9)	12·5 (8·9 to 16·8)	795·6 (611·7 to 1023·1)	4197·5 (2643·4 to 6408·6)	26·4 (19·6 to 34·3)	1128·0 (882·2 to 1425·3)	8149·7 (5832·6 to 11083·0)	127·0 (91·9 to 161·4)	2416·9 (1898·9 to 2976·0)	3048·6 (2374·1 to 3800·1)	21·9 (17·1 to 27·2)	848·2 (667·8 to 1052·3)
Tibet	1980·8 (1166·8 to 3009·3)	18·6 (10·1 to 29·5)	1041·7 (743·3 to 1376·8)	2098·2 (1285·1 to 3161·2)	23·5 (13·3 to 37·0)	1153·1 (834·1 to 1516·1)	2430·3 (1480·8 to 3569·4)	37·6 (21·2 to 58·3)	1423·3 (1025·5 to 1878·2)	3437·5 (2101·8 to 5402·5)	69·0 (40·0 to 100·7)	1906·5 (1384·4 to 2473·5)	5641·3 (3983·8 to 7804·0)	212·3 (132·1 to 303·0)	3339·9 (2465·3 to 4314·8)	2618·4 (1990·3 to 3330·3)	48·0 (28·9 to 67·6)	1464·2 (1079·1 to 1848)
Xinjiang	1416·2 (893·4 to 2047·7)	7·4 (4·5 to 12·4)	517·9 (392·7 to 669·2)	1566 (1004·8 to 2331·6)	9·8 (6·1 to 16)	596·7 (449 to 767·3)	1938·8 (1236 to 2754·7)	16·7 (11·2 to 26·3)	763·1 (590·5 to 970·6)	2929·4 (1892·8 to 4362·2)	33·8 (23·1 to 51·3)	1081·3 (839·7 to 1363·6)	4507·3 (3233·7 to 6108·4)	99·3 (69·1 to 159·9)	1856·7 (1426·4 to 2529·4)	2032·4 (1608·4 to 2541·8)	21·7 (15·7 to 33·7)	779·7 (609·6 to 997·6)
Yunnan	3422·1 (2203·7 to 4814·1)	24·1 (8·1 to 37·1)	1261·3 (760·8 to 1707·6)	3745·6 (2487·2 to 5339·2)	31·6 (10·6 to 47·1)	1472·6 (889·9 to 1964·5)	4436·3 (2900·2 to 6155·4)	58·6 (18 to 86·2)	2035·3 (1151·3 to 2680·2)	6346·3 (4197·5 to 9229·9)	109·5 (36·5 to 155·7)	2848·8 (1612·2 to 3740·3)	10480·3 (7795·9 to 13716·6)	355·9 (131·1 to 479·7)	5317·5 (2968·2 to 6722·5)	4850 (3898·5 to 5930·1)	80·1 (27·9 to 108·2)	2113·1 (1221·5 to 2718)
Zhejiang	4127·4 (2705·7 to 5714·2)	12·3 (3·6 to 19·1)	1021·6 (678·9 to 1350·3)	4754·6 (3207·8 to 6755·9)	18·2 (4·8 to 27·8)	1281·6 (835·6 to 1691)	5748·3 (3818·5 to 8012·6)	32·9 (8·5 to 49·4)	1725·7 (1081·9 to 2289·3)	8860·3 (5782 to 13004·5)	79·8 (18·8 to 119·1)	2682·5 (1539·3 to 3569·4)	18638 (13940·1 to 24392·4)	489·4 (101·7 to 697·8)	7042·5 (3354·8 to 9183·4)	7170·9 (5835·3 to 8649·8)	93·3 (20·9 to 130·8)	2258·7 (1278·7 to 2932·1)

Data in parentheses are 95% uncertainty intervals. DALY=disability-adjusted life-year.

Table 6: The percentage change of incidence, mortality and DALY rates of falls among older people aged 60 years and older by sex in mainland China and 31 provinces between 1990 and 2019

	Female			Male			Total		
Province	Change of incidence rate	Change of mortality rate	Change of DALY rate	Change of incidence rate	Change of mortality rate	Change of DALY rate	Change of incidence rate	Change of mortality rate	Change of DALY rate
Mainland China	77.0<br>(63·3 to 91·8)	57·9<br>(-32·8 to 120·1)	26·7<br>(-6·9 to 46·0)	82·9<br>(67·4 to 100·0)	63.0<br>(-22·8 to 144·5)	26·9<br>(-8·7 to 56·2)	79·2<br>(65·9 to 93·7)	59·2<br>(-24·7 to 116·4)	26·6<br>(-5·3 to 46·8)
Anhui	37·7<br>(21·3 to 56·6)	50·7<br>(-29·5 to 121·5)	6.0<br>(-20·4 to 28·3)	52·0<br>(30·1 to 80·1)	66·7<br>(-14·9 to 160·8)	14·5<br>(-16·6 to 44·6)	42·1<br>(25·6 to 61·1)	56·6<br>(-18·0 to 119·0)	10·4<br>(-15·7 to 30·4)
Beijing	90·5<br>(67·1 to 113·2)	41·9<br>(-38·6 to 113·2)	31·0<br>(9·5 to 48·4)	90·1<br>(69·4 to 114·5)	37·5<br>(-41·7 to 121)	12·7<br>(-15·3 to 36·3)	91·2<br>(70·9 to 112·6)	38·1<br>(-36·8 to 93)	21·9<br>(-1·7 to 37)
Chongqing	69·9<br>(49·5 to 89·6)	101·6<br>(-19·3 to 203)	37·0<br>(-3·2 to 68·5)	82·5<br>(58·5 to 111·1)	96·5<br>(-12·8 to 222)	34·0<br>(-8·1 to 77·4)	75·2<br>(55 to 96·3)	98·2<br>(-12·3 to 192·8)	35·3<br>(-3·8 to 67·2)
Fujian	102·8<br>(77·4 to 127·9)	80·2<br>(-37·7 to 177·4)	35·1<br>(2·2 to 70·6)	116·0<br>(87·1 to 145·3)	93·0<br>(-37 to 221·4)	41·2<br>(-10·6 to 94·0)	104·4<br>(80·6 to 127·6)	83·3<br>(-32·4 to 166·3)	36·7<br>(-2·5 to 66·7)
Gansu	33·9<br>(19·7 to 49·4)	57·0<br>(-27 to 131·3)	7·2<br>(-22·3 to 25·8)	30·3<br>(12·8 to 46·9)	44·0<br>(-19·8 to 115·6)	1·0<br>(-23·1 to 22·1)	32·3<br>(17·9 to 46)	45·7<br>(-18·8 to 105)	2·5<br>(-20·2 to 17·8)
Guangdong	117·8<br>(96·6 to 141·6)	35·9<br>(-47·6 to 109·9)	33·8<br>(1·4 to 59·3)	109·4<br>(87·2 to 140·6)	49·5<br>(-38·3 to 140·4)	26·5<br>(-11·8 to 58·3)	110·9<br>(92·8 to 132·4)	39<br>(-40·5 to 98·3)	29·9<br>(-3·4 to 52·6)
Guangxi	63·5<br>(44·0 to 82·9)	86·0<br>(-35·6 to 189·6)	28·0<br>(-17·2 to 59·1)	82·0<br>(58·5 to 105·5)	101·0<br>(-21·2 to 236·6)	37·6<br>(-12·2 to 88·0)	70·6<br>(51·8 to 88·8)	91<br>(-22·5 to 188·0)	32·3<br>(-11·8 to 66·0)
Guizhou	120·7<br>(99·9 to 138·2)	56·9<br>(-24·4 to 148·4)	52·5<br>(13·3 to 92·4)	120·8<br>(98·2 to 143·4)	64·3<br>(-23 to 167·7)	44·5<br>(-3·7 to 98·0)	121·1<br>(104·8 to 136·9)	59·7<br>(-19·5 to 134·9)	47·8<br>(7·8 to 83·2)
Hainan	27·3<br>(14·4 to 40·1)	5·1<br>(-41·1 to 73·8)	-16·3<br>(-34·9 to 4·0)	50·5<br>(32·0 to 71·7)	36·4<br>(-27·8 to 130·4)	-4·3<br>(-26·1 to 21·2)	33·1<br>(20·7 to 46·4)	9·6<br>(-26·3 to 63·1)	-12·4<br>(-25·9 to 3·2)
Hebei	76·8<br>(60·4 to 95·6)	-5·1<br>(-39·6 to 39·8)	16·8<br>(-14·2 to 32·5)	67·4<br>(53·5 to 84·9)	3·4<br>(-35·2 to 52·3)	10·6<br>(-14·4 to 30·2)	72·1<br>(59·3 to 85·8)	-2·2<br>(-32·5 to 34·5)	12·3<br>(-10·8 to 27·8)
Heilongjiang	13·5<br>(-0·6 to 27·8)	9·5<br>(-35·6 to 58·8)	-12·4<br>(-28·0 to -1·9)	22·0<br>(8·9 to 36·6)	-3·6<br>(-35·0 to 38·1)	-13·6<br>(-29·5 to -0·2)	17·6<br>(6·6 to 29·7)	-2·9<br>(-34·4 to 32·1)	-15·3<br>(-29·4 to -5·3)
Henan	94·9<br>(74·0 to 113·7)	33·8<br>(-38·7 to 101·3)	29·2<br>(-10·0 to 53·2)	96·4<br>(74·0 to 119·7)	33·9<br>(-22·4 to 99·6)	32·4<br>(-1·0 to 62·9)	95·3<br>(77·9 to 111·7)	32·3<br>(-25·2 to 83·6)	31·0<br>(-0·7 to 53·9)
Hubei	118·2<br>(97·7 to 140·1)	72<br>(-33·0 to 157·1)	49·3<br>(5·3 to 83·2)	133·5<br>(109·1 to 164·1)	91<br>(-26·6 to 213·0)	53·1<br>(-1·7 to 107·0)	123·3<br>(105·8 to 144·5)	79·9<br>(-26·3 to 161·3)	51·2<br>(4·9 to 84·8)
Hunan	36·1<br>(21·5 to 52·1)	44·6<br>(-29·7 to 110·9)	6·9<br>(-21·5 to 26·8)	72·5<br>(47·5 to 92·6)	99·8<br>(-3·6 to 213·9)	36·5<br>(-3·2 to 78·3)	50·4<br>(34·4 to 65·6)	68·4<br>(-11·2 to 135)	21·0<br>(-10·1 to 46·1)
Inner Mongolia	71·5<br>(56·1 to 87·5)	22·2<br>(-34·3 to 79·5)	15·5<br>(-17·0 to 32·1)	75·2<br>(60·3 to 91·8)	13·8<br>(-33·0 to 77·0)	11·9<br>(-17·4 to 35·5)	73·6<br>(60·2 to 87·5)	10·8<br>(-31·0 to 57·2)	9·9<br>(-16·4 to 27·3)
Jiangsu	78·8<br>(56·8 to 105·9)	107·3<br>(-40·2 to 233·4)	31·1<br>(-9·1 to 64·9)	72·1<br>(50·9 to 97·8)	92·7<br>(-31·7 to 212·4)	24·2<br>(-14·2 to 58·1)	74·6<br>(55·3 to 98·1)	97·5<br>(-33·4 to 201·3)	27·6<br>(-10·0 to 54·9)

Jiangxi	111·8 (92·0 to 134·3)	37·2 (-32·9 to 95·8)	34·2 (3·7 to 54·9)	123·5 (96·4 to 152·6)	42·6 (-31·5 to 124·1)	32 (-3·9 to 66·2)	115·9 (97·5 to 137·5)	39·4 (-28·6 to 91·5)	33·1 (0·7 to 55·1)
Jilin	21·7 (8·7 to 35·8)	24·9 (-31·2 to 73·4)	-5·8 (-25·3 to 4·3)	9·1 (-4·1 to 22·3)	-18·0 (-51·9 to 17·4)	-26·4 (-45·1 to -14·2)	14·7 (3·6 to 26·1)	-7·3 (-42·1 to 20·5)	-20·4 (-37·1 to -11·1)
Liaoning	41·7 (26·1 to 58·4)	-11·2 (-44·6 to 31·5)	-7·6 (-24·5 to 5·8)	43·7 (28·4 to 59·4)	-9·3 (-38·0 to 27·1)	-6·2 (-23·7 to 10·9)	43·2 (30·4 to 56)	-12·4 (-37·2 to 16·6)	-8·1 (-21·7 to 3·7)
Ningxia	102·1 (78·1 to 125·4)	61·2 (-30·9 to 146·8)	37·1 (-5·5 to 67)	90·6 (61·3 to 115·9)	41·9 (-32·9 to 131·2)	24·7 (-17·4 to 62·5)	99·1 (75·6 to 121·9)	46·2 (-26·9 to 113·2)	28·3 (-8·5 to 55·5)
Qinghai	42·6 (26·1 to 61·7)	88·5 (-7·2 to 172·8)	28·2 (-3·0 to 53·1)	39·9 (20·9 to 59·4)	68·6 (-10·5 to 156·4)	18·8 (-14·6 to 50)	41·3 (25·1 to 56·8)	74·4 (-5·7 to 144·2)	22·5 (-7·4 to 44·9)
Shaanxi	93·3 (76·2 to 112·3)	41·6 (-38·8 to 114·9)	32·7 (-2·7 to 61·4)	85·7 (68·6 to 103·8)	35·3 (-35·7 to 115·9)	20·1 (-18·8 to 57·6)	90·7 (76·5 to 106·9)	36·0 (-33·9 to 93)	24·1 (-10·7 to 50·8)
Shandong	49·0 (32·7 to 66·1)	23·9 (-44·6 to 87·6)	9·5 (-22·4 to 27·1)	45 (28·9 to 62·8)	19·5 (-35·7 to 86·8)	2·9 (-23·2 to 25·1)	47·0 (34·0 to 62·7)	19·4 (-35·9 to 70·7)	5·8 (-19·8 to 21·4)
Shanghai	69·4 (49·5 to 91·0)	48·5 (-25·4 to 115·1)	28·2 (9·4 to 45·2)	72·9 (52·2 to 93·3)	28·6 (-43·3 to 109·9)	13·9 (-10·4 to 37·9)	67·8 (50·4 to 86·9)	38·1 (-29·6 to 89·1)	21 (2·7 to 35·7)
Shanxi	43·8 (29·3 to 60·2)	39·1 (-33·5 to 109·0)	6·2 (-20·4 to 23·2)	38·0 (25·4 to 51·8)	18·9 (-33·7 to 79·6)	-1·8 (-25·1 to 19·0)	41·0 (29·0 to 53·4)	24·5 (-28·2 to 74·9)	0·9 (-20·5 to 17·5)
Sichuan	146·5 (121·1 to 169·5)	122·0 (-25 to 222·3)	82·5 (20·6 to 120·0)	151·2 (121·8 to 183·6)	139·9 (-5·8 to 294·7)	83·0 (17·0 to 144·2)	148·5 (125·5 to 171·4)	129·7 (-12·0 to 230·3)	82·5 (20·7 to 124·8)
Tianjin	47·9 (32·2 to 65·7)	60·5 (-38·5 to 148·1)	12·9 (-17·9 to 34·5)	53·4 (39·2 to 72·4)	31·0 (-45·3 to 109·7)	3·9 (-26·1 to 27·5)	50·4 (37·9 to 66·5)	40 (-36 to 105·2)	7·2 (-20·1 to 25·9)
Tibet	14·7 (3·4 to 26·8)	7·6 (-26·4 to 61·2)	-5·1 (-22·6 to 15·2)	27·1 (13·1 to 41·1)	-0·4 (-38·6 to 48·3)	-7·7 (-30·6 to 19·2)	18·1 (8·7 to 29)	2·1 (-23·1 to 36·5)	-6·4 (-21·3 to 10·9)
Xinjiang	39·0 (24·1 to 54)	48·4 (-3·9 to 116·1)	10·6 (-6·8 to 29·3)	31·7 (15·1 to 49·4)	42·3 (-18 to 125·5)	4·1 (-21·5 to 31·2)	37·0 (21·6 to 52·2)	40·0 (-10·7 to 109·8)	4·6 (-15·8 to 24·8)
Yunnan	122·6 (101·8 to 143·1)	100·0 (-14·8 to 180·7)	64·2 (14·5 to 98)	137·1 (111·7 to 162·4)	122·3 (-15·5 to 271·3)	71·5 (1·0 to 141·3)	128·1 (109·1 to 147·8)	109·8 (-10·3 to 198)	67·6 (12·6 to 109·3)
Zhejiang	59·1 (40·5 to 80·6)	58·8 (-43·0 to 142·0)	14·5 (-12·9 to 39·6)	78·7 (56·7 to 103·9)	73·6 (-32·7 to 186·0)	21·7 (-13·3 to 57·9)	66·2 (48·7 to 85·1)	64·8 (-35·6 to 136·9)	17·7 (-11·0 to 40·1)

Data in parentheses are 95% uncertainty intervals. DALY=disability-adjusted life-year.

Table 7: The percentage change of incidence, mortality and DALY rates of falls among older people aged 60 years and older by age groups in mainland China and 31 provinces between 1990 and 2019

	60 to 64			65 to 69			70 to 74			75 to 79			80 plus			Total		
Province	Change of incidence rate	Change of mortality rate	Change of DALY rate	Change of incidence rate	Change of mortality rate	Change of DALY rate	Change of incidence rate	Change of mortality rate	Change of DALY rate	Change of incidence rate	Change of mortality rate	Change of DALY rate	Change of incidence rate	Change of mortality rate	Change of DALY rate	Change of incidence rate	Change of mortality rate	Change of DALY rate
Mainland China	50·0 (42·1 to 59·5)	-1·6 (-50·3 to 45·1)	0·1 (-22 to 16·2)	53·0 (45·3 to 62·4)	2·8 (-47·6 to 47·9)	6·2 (-16·5 to 21·4)	52·4 (44·9 to 60·7)	12·8 (-44·9 to 61·8)	14·6 (-12·8 to 32·3)	69·5 (58·8 to 81·7)	15·9 (-41·9 to 62·9)	19·2 (-12·3 to 39·9)	123·8 (105·4 to 141·9)	51·5 (-29·5 to 115·0)	45·0 (-2·6 to 76·2)	79·2 (65·9 to 93·7)	59·2 (-24·7 to 116·4)	26·6 (-5·3 to 46·8)
Anhui	20·8 (5·1 to 34·8)	-4·6 (-56·4 to 72·6)	-12·9 (-35·3 to 10·2)	19·1 (4·2 to 33·4)	-9·1 (-54·1 to 53·2)	-11·5 (-31·1 to 6·6)	13·1 (0·6 to 27·8)	-2·8 (-51·2 to 60·4)	-6·4 (-28·1 to 12·8)	21·1 (6·8 to 40·6)	-7·6 (-48 to 47·5)	-6·5 (-26·8 to 15·5)	47·6 (31·6 to 67·5)	18·9 (-33·6 to 70·3)	9·5 (-19·7 to 34·4)	42·1 (25·6 to 61·1)	56·6 (-18·0 to 119·0)	10·4 (-15·7 to 30·4)
Beijing	49·5 (31·1 to 71·5)	-49·0 (-68·9 to -21·4)	-11·9 (-24·7 to -0·4)	60·0 (40·2 to 80·7)	-43·5 (-67·7 to -13·4)	-3·5 (-18·0 to 8·3)	53·7 (38·1 to 69·9)	-31·7 (-64·1 to 8·9)	4·9 (-14·2 to 19·2)	75·5 (56·9 to 93·6)	-19·1 (-60·1 to 33·1)	13·1 (-9·2 to 33·2)	133·5 (107·8 to 163·1)	31·5 (-39·2 to 92·0)	43 (4·7 to 71·0)	91·2 (70·9 to 112·6)	38·1 (-36·8 to 93·0)	21·9 (-1·7 to 37·0)
Chongqing	48 (29·8 to 68·1)	16 (-54·6 to 103·5)	3·8 (-25·2 to 33·3)	51·4 (32·3 to 72·0)	28·7 (-49·3 to 118·1)	12·1 (-18·7 to 41·8)	49·7 (34 to 66·9)	36·7 (-43·2 to 128·7)	19·8 (-13·9 to 52·6)	64·2 (46 to 89·5)	51·2 (-38·7 to 150·9)	30·8 (-9·7 to 70·3)	88·7 (65·8 to 112·7)	65·1 (-22·7 to 146·5)	44·7 (-2·8 to 84·0)	75·2 (55·0 to 96·3)	98·2 (-12·3 to 192·8)	35·3 (-3·8 to 67·2)
Fujian	54·3 (39·6 to 72·3)	-27·2 (-64·5 to 13·1)	-11·2 (-27·6 to 8·3)	62·7 (46·4 to 80·6)	-15·7 (-62·2 to 25·3)	-1·6 (-19·3 to 17·8)	65·3 (49·0 to 83·7)	5·7 (-58·8 to 58·1)	11·9 (-16·4 to 38·2)	90·9 (65·5 to 116·8)	23·6 (-54·6 to 91·3)	25·0 (-12·6 to 61·8)	164·1 (132·1 to 199·7)	90·3 (-35·6 to 176·9)	71·6 (5·5 to 126·2)	104·4 (80·6 to 127·6)	83·3 (-32·4 to 166·3)	36·7 (-2·5 to 66·7)
Gansu	16·8 (4·9 to 32·8)	-7 (-47·6 to 68·4)	-15·8 (-34·2 to 3·6)	14·7 (3·5 to 28·0)	-6·7 (-42·7 to 55·9)	-13·2 (-30·1 to 3·3)	11·7 (0·8 to 26·8)	4·0 (-40·4 to 77)	-6·6 (-28·5 to 13·1)	25·5 (11·0 to 42·9)	0·5 (-36·8 to 63·3)	-4·9 (-25·9 to 15·6)	58·1 (41·6 to 75·2)	26·8 (-24·8 to 100·1)	13·7 (-17·4 to 41·9)	32·3 (17·9 to 46·0)	45·7 (-18·8 to 105)	2·5 (-20·2 to 17·8)
Guangdong	66·8 (51·4 to 85·8)	-30·6 (-66·2 to 15·0)	-0·7 (-21·7 to 16·9)	73·7 (57·0 to 95·0)	-27·7 (-65·4 to 18·0)	5·3 (-16·0 to 23·3)	74·6 (58·4 to 95·7)	-14·1 (-62·5 to 38·2)	14·2 (-14·0 to 35·5)	98·4 (81·3 to 121·2)	-5·7 (-60·5 to 53·9)	19·7 (-13·3 to 48·4)	158·4 (130·1 to 191·7)	29·3 (-44·6 to 92·6)	41·8 (-3·6 to 76·6)	110·9 (92·8 to 132·4)	39·0 (-40·5 to 98·3)	29·9 (-3·4 to 52·6)
Guangxi	39·5 (22·3 to 59·2)	27·7 (-42·7 to 125·4)	2·6 (-27·1 to 36·3)	44·0 (26·7 to 62·4)	33·8 (-44 to 134·4)	8·4 (-23·3 to 40·6)	47·1 (29·1 to 66·0)	42·5 (-41·9 to 145·8)	17·9 (-19·9 to 53·4)	69·2 (45·5 to 97·8)	48·2 (-40·0 to 151·7)	27·5 (-18·2 to 68·0)	111·6 (84·8 to 139·2)	84·0 (-26·3 to 187·5)	55·9 (-7·0 to 105·4)	70·6 (51·8 to 88·8)	91 (-22·5 to 188·0)	32·3 (-11·8 to 66·0)
Guizhou	97·4 (76·5 to 119·4)	20·0 (-49·7 to 119·8)	22·7 (-14 to 66·7)	103·8 (83·2 to 127·2)	33·1 (-43 to 143·4)	34·8 (-1·2 to 80·4)	101·2 (85·1 to 122·9)	44·3 (-38·8 to 148·4)	45·5 (2·9 to 92·2)	117·3 (93·3 to 140·4)	38·9 (-33·2 to 125·2)	46·3 (5·2 to 93·2)	151 (128·1 to 173·9)	53·2 (-16·7 to 138·4)	57·2 (14·1 to 108·7)	121·1 (104·8 to 136·9)	59·7 (-19·5 to 134·9)	47·8 (7·8 to 83·2)
Hainan	7·7 (-2·9 to 22·2)	-33·2 (-60 to 14)	-32·8 (-44·1 to -18·8)	14·6 (1·7 to 28·9)	-30·7 (-55·7 to 12·8)	-28·1 (-39·4 to -15)	17·6 (4·0 to 33·0)	-20·9 (-50·1 to 38·5)	-20·7 (-34·8 to -2·8)	34·3 (18·4 to 54·3)	-14·8 (-47·8 to 52·9)	-14·2 (-31·7 to 10·7)	53·7 (38·7 to 71·2)	-1·4 (-35·9 to 66·0)	-1·6 (-24 to 27·2)	33·1 (20·7 to 46·4)	9·6 (-26·3 to 63·1)	-12·4 (-25·9 to 3·2)
Hebei	62·8 (45·7 to 84·4)	-6·7 (-40·2 to 84·9)	6·7 (-16·3 to 28·6)	63·4 (43·6 to 83·9)	-9·5 (-39·5 to 65·5)	9·8 (-10·7 to 28·9)	59·0 (41·4 to 76·2)	-10·3 (-37·6 to 53·2)	11·3 (-9·5 to 31·3)	78·8 (60·4 to 100)	-11·7 (-35·9 to 51·3)	12·3 (-10·7 to 35·9)	112 (90·9 to 137·0)	-7·2 (-30·9 to 39·7)	17·2 (-8·1 to 44·1)	72·1 (59·3 to 85·8)	-2·2 (-32·5 to 34·5)	12·3 (-10·8 to 27·8)
Heilongjiang	10·5 (-4·3 to 24·4)	-31·5 (-54·8 to 24·2)	-25·4 (-39·2 to -12)	10·0 (-1·3 to 25·2)	-31 (-53·3 to 14·8)	-22·4 (-35·2 to -10·8)	2·0 (-8·5 to 15·0)	-23·1 (-46·6 to 37·1)	-18·1 (-31·3 to -4·4)	13·0 (-0·6 to 29·6)	-19·6 (-42·7 to 35)	-15·4 (-28·8 to -0·1)	46·7 (30·1 to 66·2)	-5·2 (-30·4 to 50·3)	-4·6 (-21·8 to 14·0)	17·6 (6·6 to 29·7)	-2·9 (-34·4 to 32·1)	-15·3 (-29·4 to -5·3)
Henan	83·2 (66·6 to 103·6)	19·5 (-43·1 to 125·4)	22·3 (-11·1 to 55·0)	74·5 (53·6 to 94·5)	8·8 (-41·7 to 87·8)	22·8 (-5·4 to 47·8)	65·7 (45·2 to 89·1)	-0·6 (-40·3 to 63·7)	21·3 (-6 to 45·4)	82·5 (60·5 to 103)	-5·7 (-40·4 to 52·3)	18·6 (-8·5 to 45·1)	132·6 (107·2 to 159·2)	19·1 (-28·3 to 77·0)	32·8 (-5·5 to 65·2)	95·3 (77·9 to 111·7)	32·3 (-25·2 to 83·6)	31·0 (-0·7 to 53·9)

Hubei	87·2 (72·0 to 105·0)	15·6 (-53·7 to 98·5)	20·6 (-13·7 to 53·2)	93·9 (77·6 to 112·5)	21·2 (-49·7 to 98·6)	28·1 (-5·4 to 58·2)	95·8 (80·5 to 113·7)	34·5 (-46 to 125·1)	38·8 (-3·3 to 76·3)	117·7 (95·8 to 145·4)	34·7 (-42·7 to 115·2)	42·8 (-1·5 to 83·1)	172·8 (141·8 to 200·9)	76·8 (-26·8 to 157·2)	72·7 (8·7 to 121·9)	123·3 (105·8 to 144·5)	79·9 (-26·3 to 161·3)	51·2 (4·9 to 84·8)
Hunan	35·1 (18·6 to 53·1)	39·4 (-40·3 to 148·3)	4·5 (-24·6 to 35·2)	34·8 (19·8 to 50·7)	33·8 (-38·2 to 123·8)	5·7 (-19·7 to 31·3)	30·3 (16·1 to 42·7)	43·1 (-35·4 to 150·7)	13·5 (-17·6 to 42·9)	39·2 (23·3 to 60·9)	34·5 (-35 to 118·8)	15·0 (-16·6 to 43·8)	66·9 (49·7 to 88·9)	27·0 (-20·8 to 84·6)	19·2 (-10·1 to 47·6)	50·4 (34·4 to 65·6)	68·4 (-11·2 to 135)	21·0 (-10·1 to 46·1)
Inner Mongolia	52·7 (39·5 to 68·7)	-23·0 (-53·2 to 47·7)	-3·4 (-28·5 to 18·5)	53·4 (39·1 to 72·0)	-24·5 (-49·2 to 32·0)	-0·7 (-23·9 to 19·1)	55·2 (40·7 to 72·8)	-18·6 (-44·5 to 45·6)	3·6 (-20·1 to 26·1)	80·5 (59·7 to 101·8)	-12·9 (-39·3 to 48·2)	7·9 (-16·1 to 33)	133·3 (111·5 to 157·2)	3·5 (-27·4 to 69·0)	20·5 (-8·4 to 51·7)	73·6 (60·2 to 87·5)	10·8 (-31 to 57·2)	9·9 (-16·4 to 27·3)
Jiangsu	35·7 (20·7 to 56·7)	-6·4 (-59·3 to 61·7)	-8·9 (-27·4 to 8·6)	37·6 (22·8 to 56·4)	-4·9 (-59·6 to 60·8)	-3·9 (-22·9 to 13·0)	34·1 (19·6 to 51·7)	2·0 (-58·2 to 75·1)	2·1 (-21·1 to 23)	53·9 (35·2 to 75·7)	2·8 (-58·7 to 70·3)	6·6 (-22·4 to 31·7)	118·5 (92·2 to 150·3)	77·0 (-45·7 to 187·1)	50·4 (-13·0 to 98·6)	74·6 (55·3 to 98·1)	97·5 (-33·4 to 201·3)	27·6 (-10 to 54·9)
Jiangxi	96·6 (69·0 to 125·5)	-6·9 (-62·5 to 58·1)	9·4 (-19·4 to 35·7)	98·0 (70·2 to 127)	-4·0 (-57·6 to 57·1)	17·7 (-8 to 41·2)	92·4 (70·4 to 118·2)	7·8 (-51·5 to 68·2)	27·3 (-4·3 to 52·4)	107·2 (88·4 to 130·1)	15·7 (-43·8 to 78·5)	33·7 (-1·3 to 63·2)	139·8 (120·2 to 166·7)	33·4 (-24·5 to 92·3)	44·8 (9·2 to 77·5)	115·9 (97·5 to 137·5)	39·4 (-28·6 to 91·5)	33·1 (0·7 to 55·1)
Jilin	-0·7 (-10·6 to 15·8)	-41·1 (-63·8 to 6·5)	-32·3 (-48·3 to -20·0)	1·5 (-9·7 to 13·8)	-37·3 (-59·6 to 11·0)	-28·5 (-43·3 to -16·8)	1·5 (-8·9 to 12·0)	-33·5 (-55·3 to 11·3)	-25·0 (-39·9 to -12·7)	20·0 (5·6 to 38·6)	-26·5 (-50·8 to 24·0)	-19·7 (-36·7 to -5·0)	55·4 (35·7 to 78·0)	-11·9 (-35·4 to 33·6)	-7·5 (-27·4 to 9·6)	14·7 (3·6 to 26·1)	-7·3 (-42·1 to 20·5)	-20·4 (-37·1 to -11·1)
Liaoning	26·2 (11·8 to 39·3)	-24·8 (-51·4 to 26·6)	-15·4 (-29·4 to 0·4)	26·0 (12·4 to 38·8)	-31·6 (-53·6 to 12·5)	-15·2 (-28 to - 0·8)	23·4 (8·4 to 36·7)	-30·3 (-53·8 to 9·1)	-13·1 (-27·1 to 1·6)	41·7 (23·5 to 59·0)	-30·4 (-52·4 to 3·9)	-12·1 (-27·2 to 5·3)	90·9 (66·8 to 115·1)	-15·0 (-38·1 to 25·4)	-0·9 (-19·1 to 22·3)	43·2 (30·4 to 56·0)	-12·4 (-37·2 to 16·6)	-8·1 (-21·7 to 3·7)
Ningxia	66·8 (45·4 to 85·2)	1·1 (-51·1 to 89)	7·5 (-23·2 to 39·6)	68 (44·0 to 94·4)	1·6 (-47·0 to 82·8)	11·4 (-17·9 to 41·2)	76·5 (54·2 to 103·7)	9·8 (-42·6 to 90·2)	18·4 (-14·3 to 50·5)	108·5 (80·1 to 139·4)	20·5 (-37·4 to 104·5)	27·6 (-10·7 to 65·2)	179 (143·8 to 210·6)	46·5 (-22 to 128·4)	50·2 (2·5 to 93·7)	99·1 (75·6 to 121·9)	46·2 (-26·9 to 113·2)	28·3 (-8·5 to 55·5)
Qinghai	22·6 (8·2 to 36·5)	16·6 (-38·3 to 104·4)	1·9 (-23·5 to 27·9)	23·6 (6·3 to 42·8)	25·5 (-31·2 to 115·3)	7·2 (-17·5 to 31·9)	25·0 (9·8 to 42·3)	38·8 (-25·7 to 139·5)	15·4 (-13·8 to 43·8)	40·8 (20·8 to 62·9)	40·1 (-19·3 to 128·2)	20·2 (-8·3 to 49·7)	71·3 (52·4 to 94·2)	50·7 (-9·8 to 129·9)	33·7 (-0·4 to 68·3)	41·3 (25·1 to 56·8)	74·4 (-5·7 to 144·2)	22·5 (-7·4 to 44·9)
Shaanxi	63·4 (49·3 to 78·2)	0·0 (-57·1 to 82·7)	4·5 (-26·4 to 36·2)	70·1 (53·9 to 86·7)	4·7 (-54·1 to 74·5)	11·3 (-18 to 40·2)	73·4 (56·3 to 91·3)	11·0 (-49·5 to 87·2)	18·5 (-14·6 to 50·2)	96·3 (73·6 to 121·4)	16·5 (-44·9 to 84·1)	25·0 (-10·6 to 57·3)	138·9 (117·3 to 161·8)	32·4 (-29·6 to 87·1)	39·2 (-1·2 to 72·1)	90·7 (76·5 to 106·9)	36·0 (-33·9 to 93·0)	24·1 (-10·7 to 50·8)
Shandong	35·3 (20·6 to 54·1)	-6·0 (-53·6 to 85·8)	-5·8 (-29·1 to 13·7)	32·4 (19·1 to 51·1)	-12·8 (-53·8 to 56·8)	-4·2 (-25·8 to 11·8)	24·7 (12·2 to 39·8)	-14·2 (-53·7 to 55·2)	-2·5 (-25·8 to 15·5)	37·0 (21·8 to 56·2)	-17·2 (-52·5 to 47)	-2·4 (-26·9 to 18·3)	71·9 (53·1 to 95·4)	1·0 (-39·3 to 56·9)	9·2 (-21·8 to 33·5)	47 (34 to 62·7)	19·4 (-35·9 to 70·7)	5·8 (-19·8 to 21·4)
Shanghai	34·2 (19·9 to 49·7)	-38·8 (-67 to - 0·6)	-5·8 (-18·8 to 6·4)	40·4 (23·4 to 59·4)	-33·6 (-63·6 to 2·0)	-0·2 (-13·6 to 12·3)	36·2 (24·4 to 49·5)	-24·8 (-60·4 to 18·6)	4·3 (-11·2 to 19·6)	49·7 (35·6 to 68·4)	-23·5 (-57·3 to 15)	5·5 (-10·5 to 22·2)	110·3 (81·1 to 139·3)	34·0 (-30·7 to 86·9)	36·4 (7·3 to 62·3)	67·8 (50·4 to 86·9)	38·1 (-29·6 to 89·1)	21·0 (2·7 to 35·7)
Shanxi	30·4 (15·5 to 48)	-3·5 (-49·4 to 81·4)	-10·7 (-32 to 11·4)	31·5 (17·5 to 47·4)	-4·3 (-46·5 to 71·1)	-7·6 (-26·3 to 10·0)	24·0 (10·7 to 36·6)	-3·4 (-41·4 to 64·1)	-3·9 (-22·6 to 13·2)	36·0 (19 to 52·3)	-7·0 (-39·5 to 50·8)	-3·0 (-21·8 to 15·4)	72·1 (52·7 to 92·7)	17·8 (-26·6 to 80·2)	13·8 (-15·2 to 39·6)	41·0 (29 to 53·4)	24·5 (-28·2 to 74·9)	0·9 (-20·5 to 17·5)
Sichuan	113·3 (94·4 to 138·3)	62·6 (-42·8 to 196·3)	49·9 (1·3 to 95·9)	115·7 (94·4 to 142·2)	69·5 (-41·5 to 204·6)	57·4 (8 to 105·6)	118·5 (99·3 to 139·8)	81·1 (-37 to 208·3)	68·4 (9·8 to 118·8)	142·7 (120·9 to 169·4)	81·5 (-31·7 to 213)	74·5 (12·4 to 132·3)	181·2 (152·8 to 208·6)	97·1 (-17 to 199·9)	90·4 (19·5 to 145·5)	148·5 (125·5 to 171·4)	129·7 (-12 to 230·3)	82·5 (20·7 to 124·8)
Tianjin	25·4 (11·3 to 41·2)	-18·2 (-55·4 to 51·2)	-11·9 (-30·9 to 5·1)	26·9 (12·2 to 43·2)	-24·1 (-56·2 to 33·9)	-10·4 (-27·2 to 5·2)	31·4 (17·8 to 44·9)	-11·2 (-53·8 to 71·4)	-3 (-25·8 to 18·3)	53·8 (35·1 to 77·9)	-4·1 (-51·2 to 79·7)	4·0 (-24 to 30·5)	109 (81·3 to 142)	45·5 (-31·5 to 141·3)	35·8 (-10·1 to 74·8)	50·4 (37·9 to 66·5)	40·0 (-36 to 105·2)	7·2 (-20·1 to 25·9)
Tibet	4·1 (-6·3 to 15·4)	-29·9 (-55·9 to 13·0)	-20·6 (-39·6 to 2·0)	6·4 (-2 to 16·8)	-22·7 (-50·9 to 23·6)	-14·8 (-33·2 to 8·5)	5·2 (-4·3 to 15·1)	-15·8 (-43·7 to 36·1)	-10·1 (-28·9 to 14·9)	17·4 (2·4 to 32·0)	-12·3 (-41·3 to 33·7)	-7·7 (-27·2 to 17·5)	46·6 (33·1 to 62·9)	2·6 (-27·3 to 49·0)	2·9 (-19·6 to 31·9)	18·1 (8·7 to 29·0)	2·1 (-23·1 to 36·5)	-6·4 (-21·3 to 10·9)

Xinjiang	12·3 (-2·8 to 28·4)	-10·5 (-47·6 to 69·8)	-15·4 (-34 to 7·3)	18·0 (2·5 to 32·1)	-1·9 (-39·7 to 78·0)	-9·8 (-27·1 to 11·0)	23·9 (9·1 to 37·1)	7·5 (-30·6 to 96·8)	-2·4 (-21 to 21·6)	50·1 (33·6 to 74·1)	22·1 (-21·2 to 122·5)	8·8 (-13 to 39·5)	72·1 (56·6 to 92·5)	26·2 (-7·1 to 100·1)	19·7 (-0·6 to 50·5)	37·0 (21·6 to 52·2)	40·0 (-10·7 to 109·8)	4·6 (-15·8 to 24·8)
Yunnan	102·6 (83·7 to 126)	54·7 (-42·4 to 166·3)	36·7 (-9·3 to 83·4)	104·9 (82·6 to 129·8)	55·9 (-39·5 to 158·1)	42·9 (-1·5 to 85·1)	103·1 (84 to 124)	79·6 (-33·3 to 180·8)	60·6 (4·3 to 108·2)	120 (95·9 to 144·9)	73·8 (-30·3 to 169·1)	64·1 (7·7 to 113·5)	152·6 (128·5 to 180·0)	89·3 (-9·5 to 171·6)	78·1 (17·6 to 128·9)	128·1 (109·1 to 147·8)	109·8 (-10·3 to 198·0)	67·6 (12·6 to 109·3)
Zhejiang	27·4 (13·9 to 44·9)	-21·7 (-63·4 to 22·9)	-18·8 (-32·4 to -2·7)	34·5 (16·9 to 52·0)	-12·8 (-63·1 to 32·9)	-11·2 (-26·6 to 4·4)	33·4 (18·9 to 47·7)	-6·7 (-61·7 to 42·9)	-4·6 (-22·3 to 14·0)	50·5 (32·1 to 71·5)	1·2 (-60·1 to 51·2)	2·9 (-21·3 to 25·0)	110·5 (82·7 to 144·8)	58·9 (-41·2 to 126·9)	39·8 (-4·2 to 75·7)	66·2 (48·7 to 85·1)	64·8 (-35·6 to 136·9)	17·7 (-11 to 40·1)

Data in parentheses are 95% uncertainty intervals. DALY=disability-adjusted life-year.

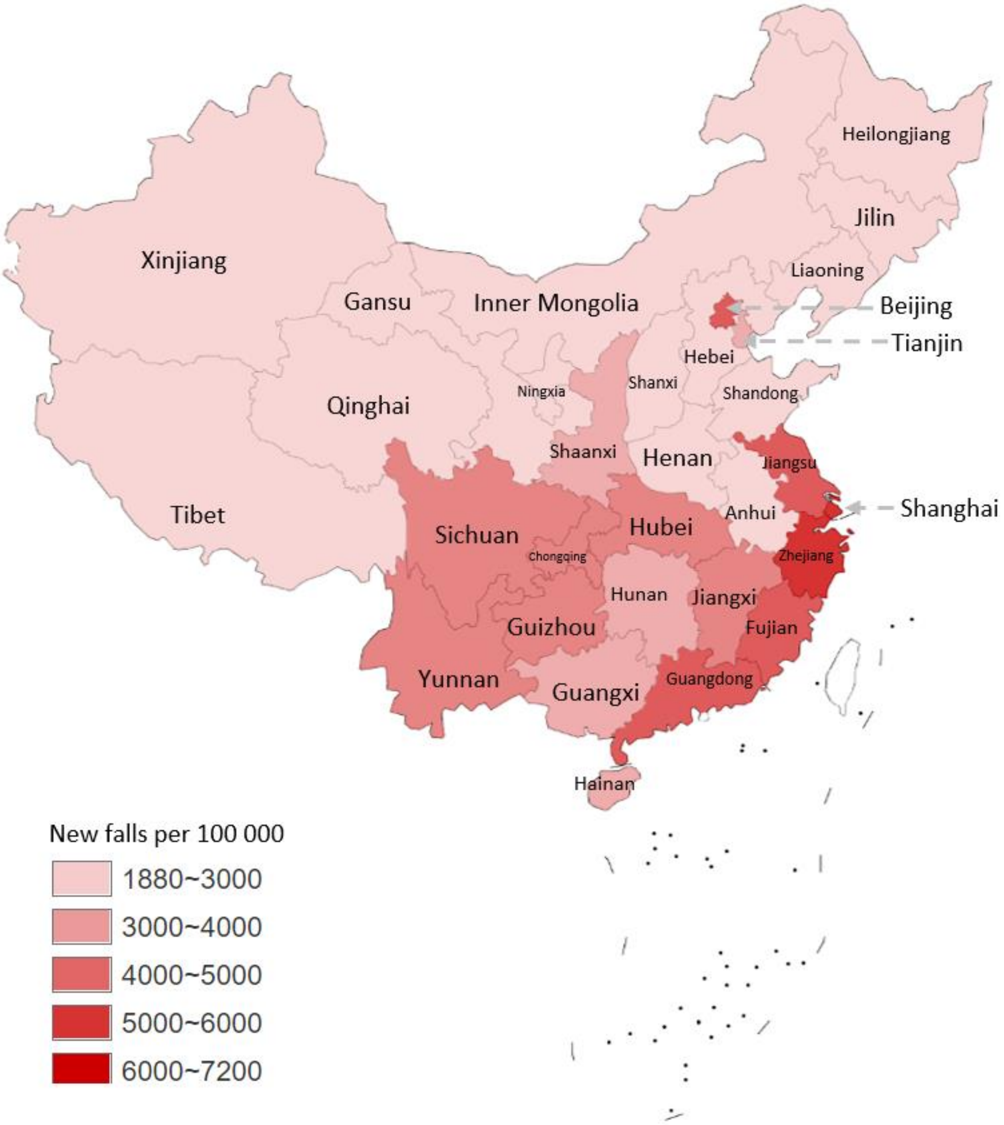

Figure 1: The incidence rate of falls among older people aged 60 years and older in 31 provinces of mainland China in 2019

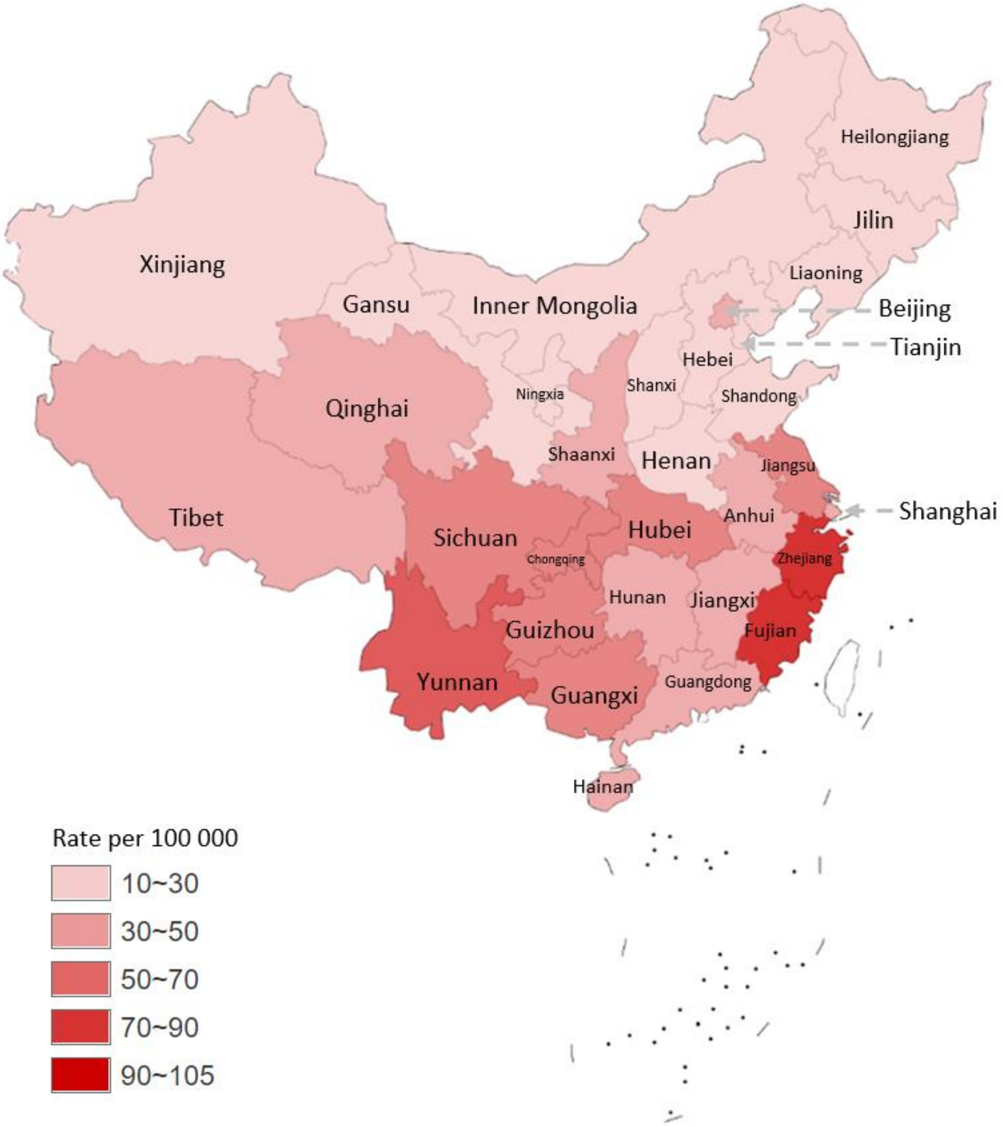

Figure 2: The mortality rate of falls among older people aged 60 years and older in 31 provinces of mainland China in 2019

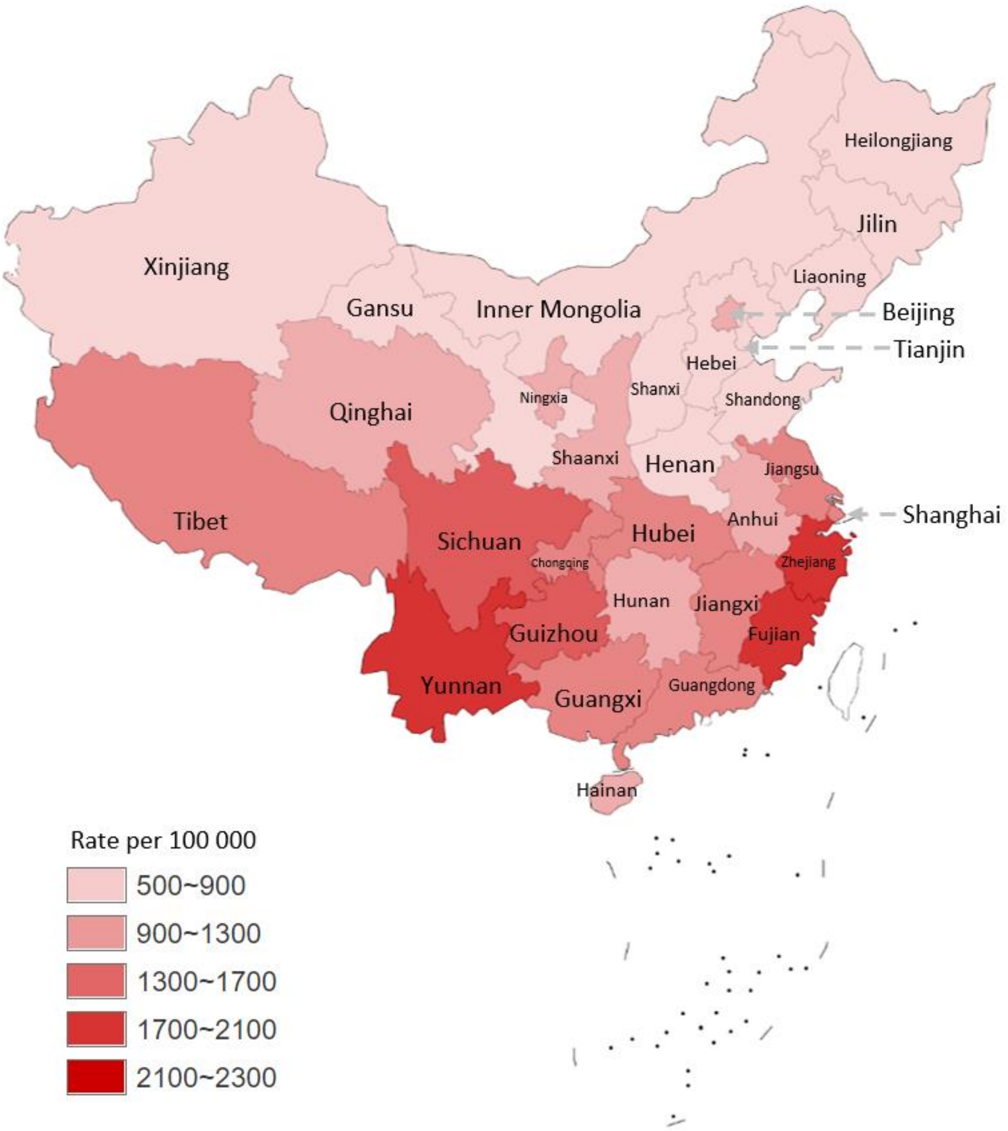

Figure 3: The DALY rate of falls among older people aged 60 years and older in 31 provinces of mainland China in 2019
DALY=disability-adjusted life-year.

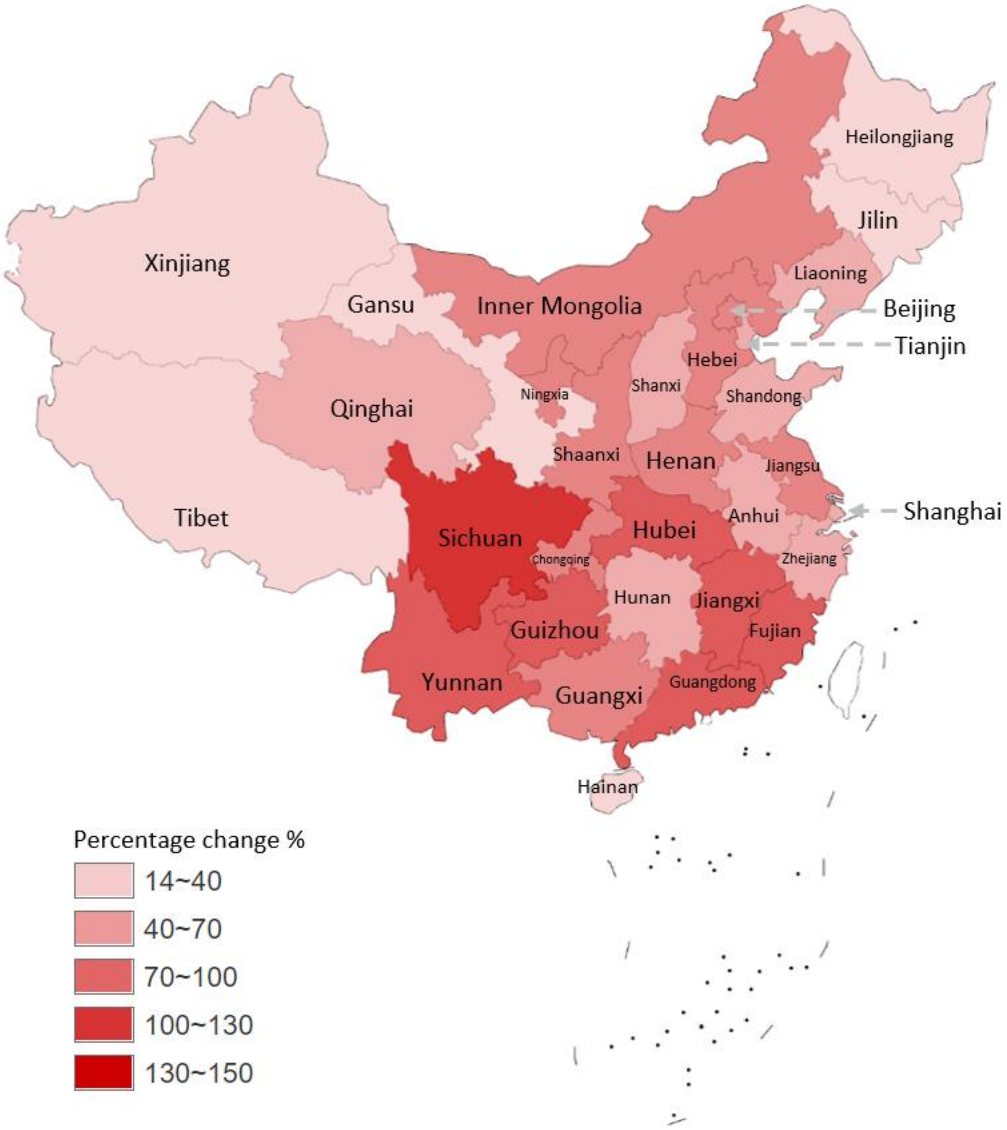

Figure 4: The percentage change of incidence rate of falls among older people aged 60 years and older in 31 provinces of mainland China between 1990 and 2019

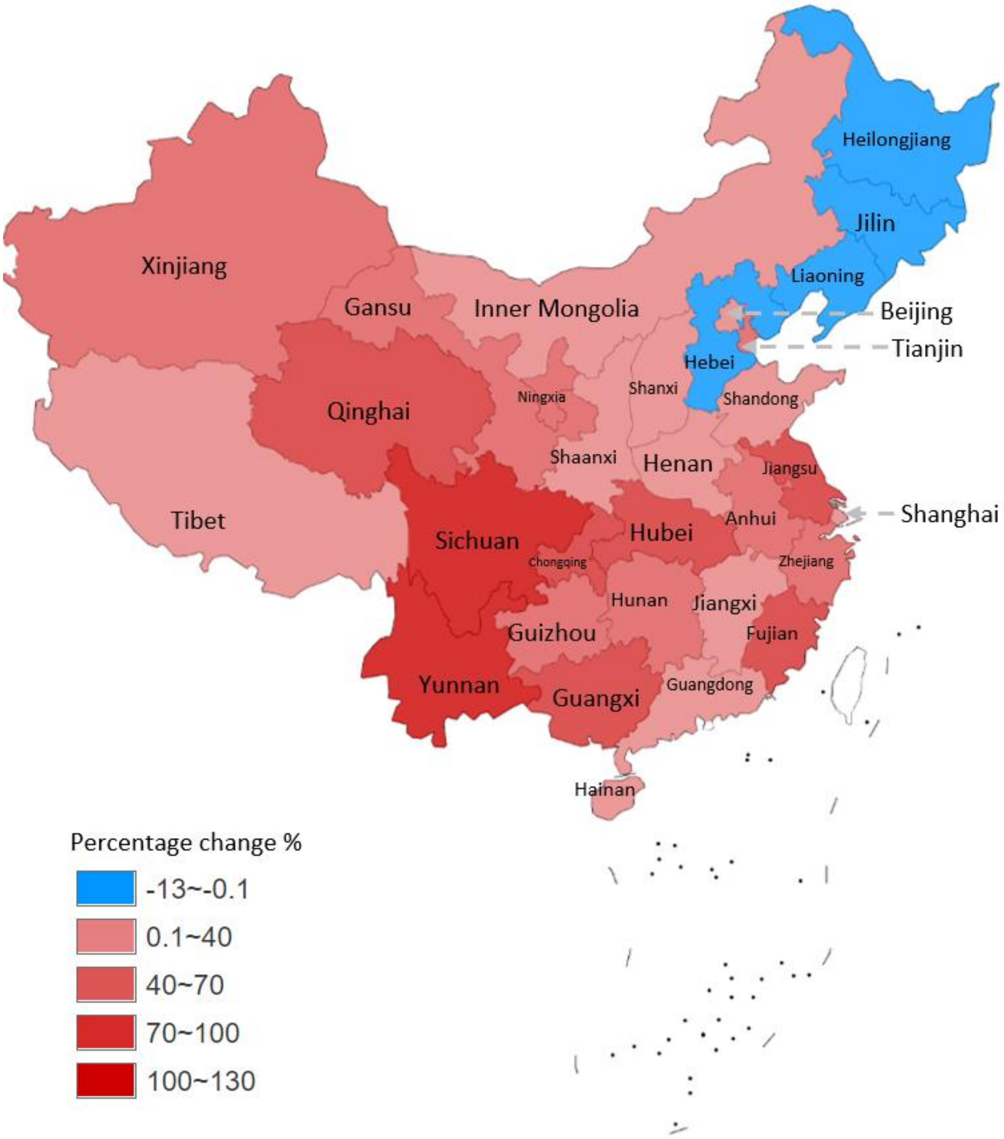

Figure 5: The percentage change of mortality rate of falls among older people aged 60 years and older in 31 provinces of mainland China between 1990 and 2019

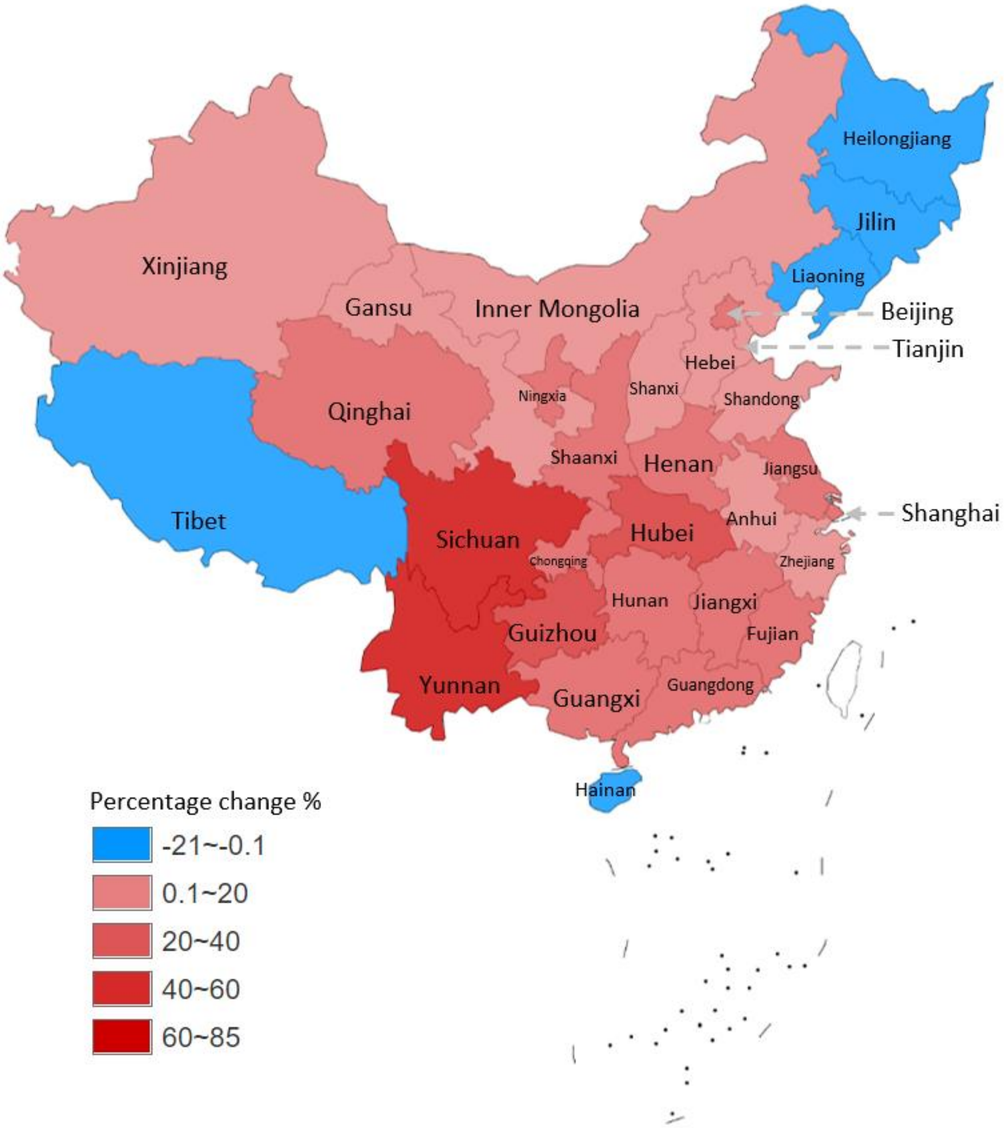

Figure 6: The percentage change of DALY rate of falls among older people aged 60 years and older in 31 provinces of mainland China between 1990 and 2019
DALY=disability-adjusted life-year.
